# Affordable Non‐Invasive Machine‐Aided Phenotyping Identifies Phenotypic Variation to Soil Stress Across the *Arabidopsis thaliana* Life Cycle

**DOI:** 10.1111/ppl.70427

**Published:** 2025-08-07

**Authors:** Marie Christin Knopf, Petra Bauer

**Affiliations:** ^1^ Institute of Botany Heinrich‐Heine‐University Düsseldorf Germany; ^2^ Cluster of Excellence on Plant Science (CEPLAS) Heinrich‐Heine‐University Düsseldorf Germany

**Keywords:** alkaline calcareous soil, leaf chlorosis, life cycle, machine‐aided phenotyping, rosette

## Abstract

*Arabidopsis thaliana* is a model species for uncovering genetic adaptation to alkaline calcareous soils (ACS). This species thrives in ACS, often occurring in dry marginal and urban environments. Existing research largely focused on vegetatively grown seedlings, with a notable lack of studies examining phenotypic variations across the life cycle. A valuable tool for understanding stress resilience is machine‐aided phenotyping, as it is non‐invasive, rapid, and accurate, but often unavailable to small plant labs. Here, we established and validated an affordable multispectral machine‐aided phenotyping approach implementable by individual labs. We collected and correlated quantitative growth data across the entire plant life cycle in response to ACS. We used an *A. thaliana* wildtype and the coumarin‐deficient mutant *f6'h1‐1*, exhibiting chlorosis under alkaline conditions, to assess weekly morphological and leaf color data, both manually and using a multispectral 3D phenotyping scanner. Through correlation analysis, we selected machine parameters to differentiate size and leaf chlorosis phenotypes. The correlation analysis indicated a close connection between rosette size and multiple spectral parameters, highlighting the importance of rosette size for growth of *A. thaliana* in ACS. The most reliable phenotyping was at the beginning of the bolting stage. This methodology is further validated to detect novel leaf chlorosis phenotypes of known iron deficiency mutants across growth stages. Hence, our affordable machine‐aided phenotyping procedure is suitable for high‐throughput, accurate screening of small‐grown rosette plants, including *A. thaliana* , and enables the discovery of novel genetic and phenotypic variations during the plant's life cycle for understanding plant resilience in challenging soil environments.

## 1 Introduction

More than 30% of the earth's soils are alkaline and/or calcareous (Chen & Barak, Chen and Barak 1982), and the proportion may rise with global warming and by human activities (Rengel 2011; Sun et al. 2023). These soils impose several challenges for plant growth (Taalab et al. 2019), and hence it is important to understand which genetic factors help plants to better thrive under these conditions.

In calcareous soils, calcium carbonate (CaCO_3_) determines the main soil properties. Such soils are formed over calcareous parent rocks or by irrigation with carbonate‐rich water and often occur in arid or semiarid regions (Wahba et al. 2019). The pH is typically high and buffered by carbonates between 7.5 and 8.2 (Maulood et al. 2012; Wang et al. 2020). Besides calcium (Ca), sodium (Na) is a relevant ion in calcareous soils and is additionally related to alkalinization (Hu et al. 2021). Soils in urban environments are typically alkaline and saline, especially in industrial and traffic areas, but also near residences (Horváth et al. 2015; Yang and Zhang 2015). Hence, model plant species growing naturally in urban and natural sites on alkaline and saline soil conditions can be highly informative for uncovering stress resilience mechanisms. Plant adaptation to urban environments is increasingly important due to the rise in urban populations and urbanized areas and the potential of urban ecology and gardening to positively affect human well‐being, food security, and greenhouse gas emission reduction (McDonnell and MacGregor‐Fors 2016; Ferreira et al. 2018; Tomatis et al. 2023; Zhang et al. 2023). Above all, *Arabidopsis thaliana* is the most widely used model plant species and a reference to many crops and wild plant species. It inhabits many edaphically different habitats, including urban, alkaline, and saline environments, indicating it has adaptive genetic mechanisms (Terés et al. 2019; Pérez‐Martín et al. 2022; Schmitz et al. 2024). Although *A. thaliana* has been widely studied, there are still numerous open questions with regard to genetic mechanisms and genetic variation contributing to the success of this plant species in thriving in disturbed urban sites.

Plants may lack iron (Fe) in alkaline calcareous soil (ACS) conditions as the bioavailability of Fe is low under high pH (Vélez‐Bermúdez and Schmidt 2023). *A. thaliana* iron homeostasis mutants often have stronger leaf chlorosis phenotypes than wildtypes on ACS (Long et al. 2010; Schmid et al. 2014; Zhang et al. 2015; Li et al. 2016; Lei et al. 2020). A key regulator for Fe acquisition is the essential FER‐LIKE IRON DEFICIENCY‐INDUCED TRANSCRIPTION FACTOR (FIT). The severely growth‐compromised loss‐of‐function mutant *fit‐3* only grows upon Fe fertilization (Jakoby et al. 2004; Schwarz and Bauer 2020). One of the FIT target genes encodes FERULOYL‐COA 6*'*‐HYDROXYLASE1 (F6'H1) catalyzing the first enzymatic step in coumarin biosynthesis (Schmid et al. 2014). Coumarins are important secondary compounds that allow *A. thaliana* to mobilize Fe and grow on ACS (Schmid et al. 2014; Terés et al. 2019; Gautam et al. 2021; Robe et al. 2021). *f6'h1* loss of function mutant plants have slight Fe deficiency and leaf chlorosis in alkaline conditions (Schmid et al. 2014; Robe et al. 2021). POPEYE (PYE) is another transcription factor (Long et al. 2010), and the E3 ligases BRUTUS‐LIKE1 and BTSL2 interact with transcription factors related to PYE and affect Fe utilization negatively (Rodríguez‐Celma et al. 2019; Lichtblau et al. 2022). Loss‐of‐function mutants of *pye* and *btsl1 btsl2* do not show leaf chlorosis but complete their life cycles in turf soils without Fe fertilization. Their phenotypes become visible in Fe‐limited or resupply conditions (Long et al. 2010; Rodríguez‐Celma et al. 2019; Stanton et al. 2023). Many studies on Fe homeostasis in plants focused primarily on the investigation of seedlings on agar plates or in hydroponic solutions containing low Fe concentrations (e.g., Nguyen et al. 2022; Tabata et al. 2022) or they investigated germination on ACS (Wala et al. 2022).

Plant phenotyping is a powerful method to discriminate genetic diversity across the life cycle, particularly using non‐invasive automated methods (Arvidsson et al. 2011; Vasseur et al. 2018). This has been performed in large high‐throughput phenotyping pipelines including automatic moving and watering of the plants (Granier et al. 2006; Arend et al. 2016; Pieruschka and Schurr 2019). These platforms enable non‐destructive, fast, standardized, and repeated measurements of single plants over time and can replace tedious manual plant phenotyping and destructive measurements (Poorter et al. 2023). In multispectral scanning, 3D laser scanning provides physical information of an object and positions, while spectral analysis gives hints on plant characteristics like chlorophyll content, nutrient status, and water stress (Xia et al. 2023). The laser and near‐infrared measurements do not affect photosynthetic performance of the scanned plants (Kjaer and Ottosen 2015). Despite the advantages of large phenotyping facilities, less automated and smaller lab‐based imaging devices would be of great benefit to small individual research units. They are affordable solutions, available for restricted budgets and spaces and can be flexibly used. One commercially available, non‐destructive phenotyping device is the PlantEye (Vadez et al. 2015) offered in the small and portable format of a MicroScan (Phenospex, Heerlen, The Netherlands). It is an environmental light‐independent 3D multispectral imaging system. However, a standardized procedure to use the MicroScan for studying stress resilience across the life cycle in the model species *A. thaliana* does not yet exist. Despite a number of studies on growth of *A. thaliana* in ACS conditions, the phenotypes determined were mainly manually measured phenotypes (Long et al. 2010; Schmid et al. 2014; Terés et al. 2019; Gautam et al. 2021). In our view, there is no systematic study addressing the accuracy of machine phenotyping for small rosette plant species like *A. thaliana* in stressful environments, causing leaf chlorosis, such as ACS. The PlantEye was used in studies of plant or plant community performance (Manavalan et al. 2021; Li et al. 2022; Yang et al. 2022; Gedif et al. 2023; Zieschank and Junker 2023). *A. thaliana* was to our knowledge only used in one of them (Yang et al. 2022), but no validation of the approach was shown. It is not clear whether machine phenotyping with a PlantEye device discriminates weak leaf chlorosis phenotypes of *A. thaliana* as they occur in ACS.

Several challenges need to be solved when establishing machine‐aided phenotyping for discriminating genetic diversity in ACS conditions in *A. thaliana* . One challenge with automated multispectral phenotyping is that the non‐plant background must be excluded from analysis (Arvidsson et al. 2011; Li et al. 2014; Vasseur et al. 2018), so that plant leaf color shades can be distinguished (Matsuda et al. 2012; Ochogavía et al. 2014; Dobbels and Lorenz 2019). This is a particular challenge when small plants are to be grown, especially those developing a rosette of leaves close to the soil surface, as is the case in *A. thaliana* . Moreover, a suitable condition close to natural calcareous soil conditions must be imitated in the lab to screen for genetic diversity involving transgenic lines (Msilini et al. 2009; Schmid et al. 2014; Murgia et al. 2015; Ben Abdallah et al. 2017; Terés et al. 2019; Ding et al. 2020; Gautam et al. 2021; Rosenkranz et al. 2021; Pérez‐Martín et al. 2022; Busoms et al. 2023). Much of the published phenotypic screening in *A. thaliana* has been conducted at the seedling or early vegetative stage (Schmid et al. 2014; Satbhai et al. 2017; DeLoose et al. 2024), precluding observations of phenotypic diversity during reproduction. Hence, not all Fe deficiency phenotypes have been fully exploited. Machine‐driven phenotyping would clearly be a benefit. The MicroScan system with the PlantEye device is affordable and hence is a small, portable, and cost‐effective solution for plant phenotyping in a single lab setting. While not fully automated, the system allows rapid and frequent plant scanning, enabling time course data collection over the life cycle of individual plants with minimal effort.

This study aimed to establish an affordable innovative high‐throughput machine‐aided phenotyping procedure using the PlantEye suited to detect genetic variation in standard laboratory ACS stress resilience experiments in a non‐destructive manner throughout the life cycle of *A. thaliana* . Being able to apply machine phenotyping throughout the plant life cycle will be useful for uncovering novel genetic diversity for adaptation in *A. thaliana* to stress conditions like ACS. Such findings can explain the success of this species in urban environments and be informative for translational research to crops.

## 2 Materials and Methods

### 2.1 Plant Material

Lines of *A. thaliana* (L.) Heynh. were multiplied in parallel at the Heinrich Heine University. They were wildtype (WT, Col‐0) and four mutants in the Col‐0 background, *f6'h1‐1* (Schmid et al. 2014), *fit‐3* (Jakoby et al. 2004), *pye‐1* (Long et al. 2010), and *btsl1 btsl2* (Rodríguez‐Celma et al. 2019).

### 2.2 Plant Growth Conditions

A detailed description of plant growth is provided in the Supporting Information Methods and Figure S1. Three plant growth experiments were conducted (Figure S1A,B). Briefly, seeds were surface‐sterilized with a solution containing 6% NaOCl and 0.1% TritonX100, stratified in darkness at 4°C, and germinated on half upright Hoagland plates for 8 days (16 h light, on average 135 μmol m^−2^ s^−1^ (fluorescent tube light, ecolux F17 T8 17W 4100K), 21°C in light, 19°C in darkness and 50% relative humidity, CU‐36L4/D, CLF Plant Climatics). On the eighth day, plants were transferred to soil. Pots filled with soil were covered with matt blue vinyl foil, leaving a hole for plants to grow (Figure S2A). Plants were grown in plant cabinets with 16 h light (98–112 μmol m^−2^ s^−1^, Polyklima True Daylight + LED), 21°C during light, and 19°C during darkness. Humidity was not controlled. Eight pots fitted in one tray. Soil was prepared with a peat substrate supplemented with different amounts of CaCO_3_ (AppliChem), NaHCO_3_ (Fisher Scientific), and sand according to Figure S2B and as specified in the text, named control, ACS1‐5, and ACS3‐25% and −50% sand. In short, for ACS 1–5, 6, 8, 8, 31.6, and 30 g of CaCO_3_ and 3, 4, 4, 13.7, and 20 g of NaHCO_3_, respectively, were added per liter peat‐based soil. For ACS3‐25% sand and ACS3‐50% sand, the respective percentage of sand by volume was mixed with ACS3 soil. Control soil was peat‐based soil without the addition of CaCO_3_, NaHCO_3_, or sand. Plants in ACS1 and ACS3 were watered with NaHCO_3_ solution. For details on watering, see Figure S2B and Supporting Information Methods. The pH was determined according to the procedure below. In Experiment 3, the plants were moved to a walk‐in growth chamber with 16 h light (80–120 μmol m^−2^ s^−1^, BX120c4, VAYOLA), 21°C day temperature, 19°C night temperature, and 57% humidity after 3 weeks in soil instead of being kept in a growth cabinet. Sixteen plants were grown per line and condition for Experiments 1 and 2, eight for Experiment 3 (Figure S1A,B). Trays were regularly rotated.

### 2.3 Soil pH Determination

Deionized water was added to 15 g wet soil (5 g soil dry weight) to reach 50 mL in a Falcon tube, rotated for 30 min with 20 rotations per min, and centrifuged for 10 min at 4000 *g* and 20°C (Heraeus Multifuge X1R, Thermo Fischer Scientific). The supernatant was filtered through a paper filter (Folded filters, 322345, Schleicher & Schuell), and the pH was determined with a pH electrode (S20 Seven Easy, Mettler Toledo). An average was calculated from two samples per condition.

### 2.4 Destructive Determination of the Chlorophyll Content

The chlorophyll content was determined from whole rosettes of plants. The rosettes were frozen ground in liquid nitrogen, and ~100 mg of plant material was used and weighed before acetone extraction. After centrifugation at 15000 *g* for 10 min, the absorption was measured at 470, 642, and 662 nm (Shimadzu UV visible Spectrophotometer UVmini‐1240 and Hellma OS 104‐OS cuvette). The pigment contents per fresh weight were calculated according to the following formulas (Lichtenthaler 1987): Chlorophyll *a*: (μg ml^−1^) = (11.24*A662–2.04*A642) * dilution; Chlorophyll *b*: (μg ml^−1^) = (20.13*A642–4.19*A662) * dilution; Carotenoids: (μg ml^−1^) = [(1000*A470‐1.90*Chla—63.14*Chlb)/214] * dilution.

### 2.5 Manual Morphological Phenotyping

Plants were photographed (α 6000, Sony) weekly. Different phenotypical parameters were manually determined either using the photos and performing measurements with ImageJ or by directly measuring the plants. Manual measurements were performed to determine the following parameters: the rosette diameter (cm), manual rosette area (mm^2^), manual rosette convex hull (mm^2^), plant fresh weight (mg), rosette fresh weight (mg), plant height (cm), number of siliques, number of side branches, and flowering time (days after sowing, DAS) as outlined in Figure S3 and described in the Supporting Information Methods. The plant fresh weight and rosette fresh weight were determined destructively using a scale (Secura225D, Sartorius Lob Instruments GmbH & Co. K). Whole plants were scanned with the PlantEye; then shoots were removed, and the remaining rosettes were scanned again. Afterwards, the shoots and rosettes were immediately weighed to prevent water loss before weighing. For whole plant fresh weight, the weight of shoots and rosettes was added for each plant. Plants used here were the same plants as those used for pigment content analysis and were afterwards no longer included in any measurements.

### 2.6 Machine (PlantEye) Phenotyping

Machine phenotyping was performed with a MicroScan device with PlantEye F600 (Phenospex, Heerlen, The Netherlands) and the implemented software HortControl (Phena version 2.0, HortControl version 3.85). Automated phenotyping requires improved techniques to exclude non‐plant background from the analysis (Arvidsson et al. 2011; Li et al. 2014; Vasseur et al. 2018). Plant pots were therefore covered with blue foil and measured one by one by placing the pots in a blue holder that kept the plants at a fixed height and within the measured unit. Each plant was either measured four times (Experiments 1 and 2) or twice (Experiment 3) at each time point for technical replication of the measurements, except for the pigment extraction in Experiment 2, for which only one measurement was done. Plant pots were turned by 90° between the repeated measurements. The color hue range from values between 200° and 360° (blue to purple) was removed from all images that were used for calculations to remove the non‐plant background and only use the plant areas for analysis. The PlantEye parameters were split into morphological parameters (Figure S4) and spectral parameters (Figure S5). Two of the morphological parameters describe the area of a plant. The 3D leaf area is the three‐dimensional area of a plant in mm^2^, and the projected leaf area is the area covered by a plant seen from the top view in mm^2^. Three of the morphological parameters are linked to plant height, namely the canopy light penetration depth, the plant height max, and the plant height averaged, all in mm. The canopy light penetration depth indicates how deep the laser reaches into the plant canopy, and the plant height max is the distance between the pot height and a plant's highest point. In the case of the plant height averaged, the highest point is replaced by the average of the highest 10% of points. The digital biomass is the product of plant height averaged and 3D leaf area. Several of the morphological parameters focus on the convex hull, a convex two‐dimensional polygon drawn around the plant area. Among those are the convex hull area in mm^2^, the convex hull circumference in mm, and its maximum width in mm. The convex hull area coverage is the percentage of the convex hull covered by the plant, while the convex hull aspect ratio is the quotient of the convex hull maximum width and the perpendicular line to it at its midpoint. The last two morphological parameters are the surface angle average identifying the average angles of all triangles forming a plant and the voxel volume total in mm^3^, estimating a plant's volume. Using non‐invasive phenotyping methods like the PlantEye, plant leaf color shades can be distinguished (Matsuda et al. 2012; Ochogavía et al. 2014; Dobbels and Lorenz 2019). The spectral parameters include the three values hue, saturation, and lightness of the HSL color space, with hue defining the color in°, the lightness ranging from 100% for white to 0% for black, and the saturation with 0% in the case of grey and 100% for bright colors. Further spectral indices are calculated by the reflection of different colors. These include the greenness leaf index (GLI) calculated using the reflection in green, red, and blue: (2·GREEN−RED−BLUE)/(2·GREEN + RED + BLUE) and the normalized pigment chlorophyll index (NPCI) relying on red and blue: (RED−BLUE)/(RED + BLUE). The other two indices additionally include reflection in near‐infrared. The normalized difference vegetation index (NDVI) is calculated by the following formula: (NIR−RED)/(NIR + RED) and the plant senescence reflectance index (PSRI) by this one: (RED−BLUE)/NIR. For all these parameters, the average per plant and the percentage of voxels within different definable ranges were calculated in the HortControl software implemented in the PlantEye using the pre‐set values.

### 2.7 Statistical Analysis

For the comparison of several groups, two‐way ANOVA and Tukey tests were performed in R (R 4.4.0). Figures were prepared in the Statistical Package for Social Science (SPSS, International Business Machines Corporation, version 29.0.0.0, license version 5725‐A54) or Microsoft Office 2016 Excel. Correlation analysis was done in SPSS. After testing for normal distribution (Kolmogorov–Smirnov) Spearman‐Rho correlation and graph plotting were done in SPSS. Hierarchical clustering was done in R (R 4.4.0). The data were split between time points to avoid disturbance of the analysis by unavailable data. Data were z‐score transformed by phenotypic parameters (scaler function, liver package, R). Then graphs were created using the functions dist (method: euclidean) and hclust (agglomeration method: complete; stats package). *N* indicates the number of individual plants, except in the case of correlation analysis of weekly measured manual traits (rosette diameter in cm, manual rosette area in mm^2^, manual rosette convex hull in mm^2^). Here *N* was the number of collected data points, including repeated measurements of single plants.

## 3 Results

### 3.1 Correlation Analysis of Manual and Machine‐Derived Phenotypic Parameters for Plant Growth on ACS

First, we set out to validate the machine‐aided MicroScan system (PlantEye, Phenospex) for its applicability for small rosette plant species and its effectiveness in high‐throughput phenotyping under ACS conditions. We assumed that specific manually measured phenotypic parameters previously recorded in ACS could be replaced by PlantEye parameters, offering the many advantages associated with machine phenotyping, such as increased objectivity, efficiency, and reduced labor intensity (Akhtar et al. 2024). At first, we collected suitable manual and machine‐derived plant growth data for *A. thaliana* wildtype (WT) and its coumarin‐deficient mutant *f6'h1‐1* under control and up to seven ACS conditions, representing a range of pH values from pH 6.2 (control) up to in between 7.8 (intermediate ACS) and 8.3 (severe ACS; details in Figure S2B). Data were recorded throughout the growth cycle in two independent experimental series. They showed that the *f6'h1‐1* mutant indeed suffered more severe iron deficiency and chlorosis than the wildtype under alkaline conditions (overview in Figure S1A; all plant growth phenotyping data in Table S1, details on parameters in Figures S3–S5). This has been expected (Schmid et al. 2014). We also confirmed it in an additional experiment (Figure S6), showing that growth in ACS conditions was compromised. Plants had a reduced size, whereby *f6'h1‐1* plants had more intense visible leaf chlorosis than WT, as revealed by manual measurements of the rosette diameters and SPAD values.

**FIGURE 1:**
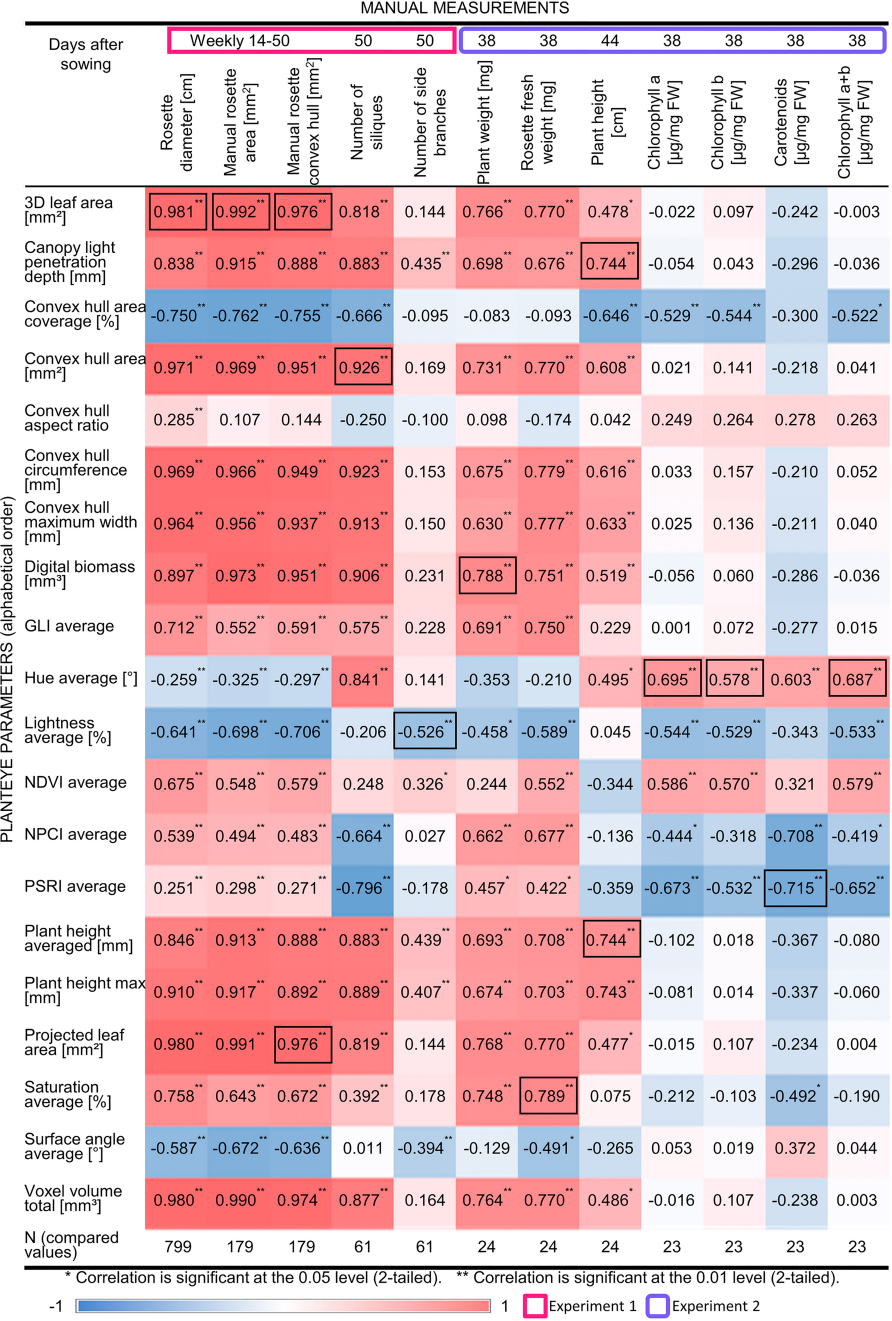
Correlation analysis of manual and machine‐derived phenotypic parameters. Data of twelve manual and 20 machine‐derived PlantEye parameters for *Arabidopsis thaliana* wildtype (WT) and its coumarin‐deficient mutant *f6'h1‐1* under control and up to seven alkaline calcareous soil (ACS) conditions, representing a scale of differing pH values from pH 6.2 (control) up to 8.3 (severe ACS), were recorded during two experiments and subjected to correlation analysis. Data were collected at the indicated time points. Rectangles around time points indicate two experiments (pink: Experiment 1; lavender rounded corners: Experiment 2). As indicated, either rosettes (pigment content, rosette fresh weight) or whole plants (remaining parameters) were used. Spearman Rho analysis was conducted in SPSS (*p < 0.05, **p < 0.01). Heatmap color codes for correlation coefficients (−1 blue, 0 white, +1 red). The strongest correlation per manual parameter is marked with a box. *N* = number of data points as indicated.

Then, we used the obtained growth data and conducted a correlation analysis between 12 manually measured and 20 PlantEye‐derived parameters. We detected many correlations in the datasets (Figure 1), which was a strong hint of the accuracy of the phenotyping procedure. Very importantly, each of the manual parameters matched with at least four or more significantly correlating PlantEye values. Key findings included the correlation of manual parameters like rosette diameters, plant height, plant weight, and chlorophyll content with their PlantEye equivalents like 3D leaf area, plant height max, digital biomass, and spectral parameters including hue average, PSRI average, NDVI average, and lightness average (Figure 1). Surprisingly, one manual parameter, namely the rosette diameter, significantly correlated with all 20 PlantEye parameters. All manual parameters related to rosette size, in addition to rosette diameter, also manual rosette area, manual rosette convex hull, and rosette weight, correlated best with 20, 19, 19, and 17 PlantEye parameters. This finding speaks in favor of the importance of the rosette size for *A. thaliana* growth physiology and development in ACS.

**FIGURE 2:**
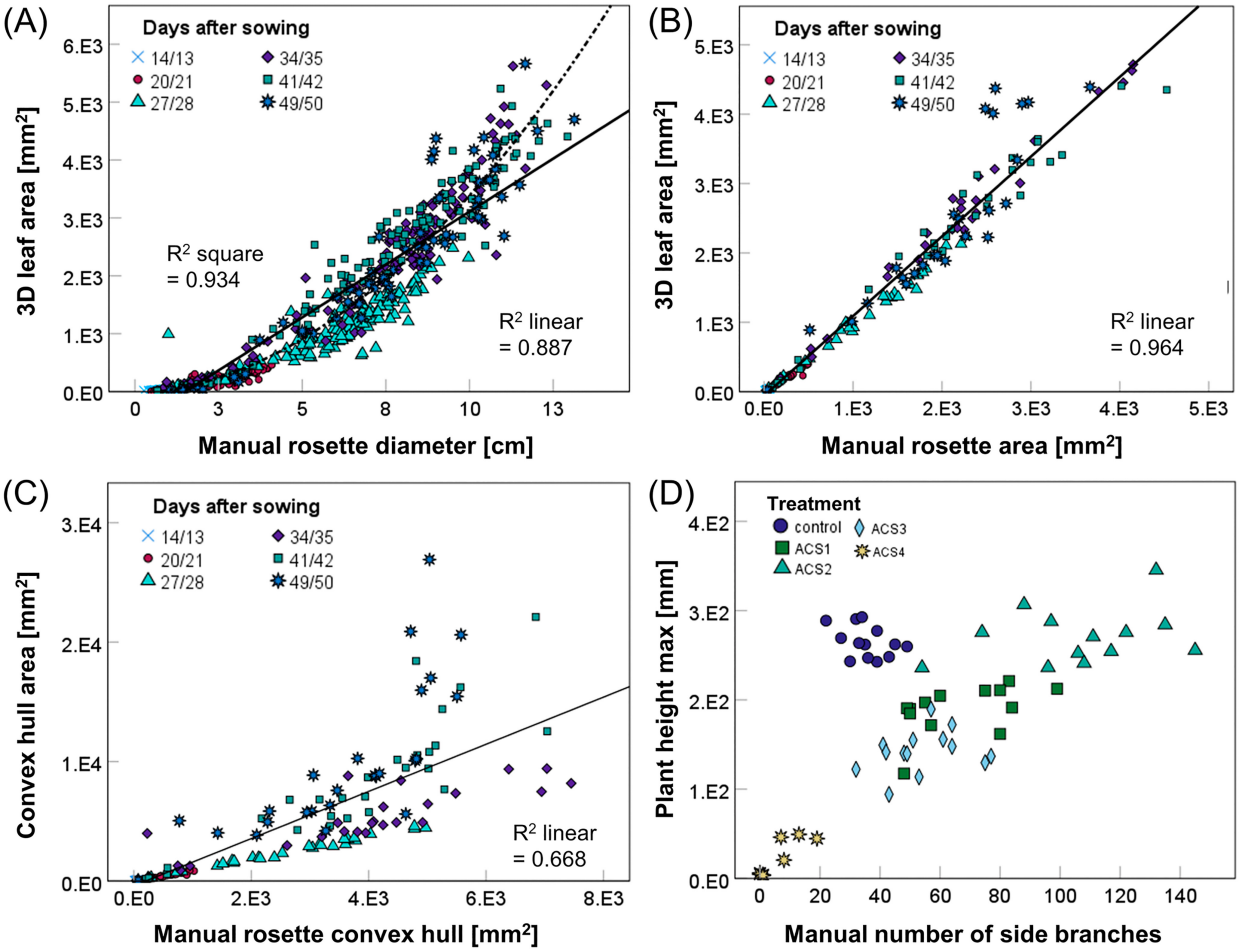
Scatterplot analysis of selected manual and machine‐derived phenotypic parameters. Scatter plots of (A) rosette diameter with PlantEye 3D leaf area (*N* = 799 data points), (B) manual rosette area with PlantEye 3D leaf area (*N* = 179 data points), and (C) manual rosette convex hull with PlantEye convex hull area (*N* = 179 data points). Growth data for *Arabidopsis thaliana* wildtype (WT) and its coumarin‐deficient mutant *f6'h1‐1* under control and alkaline calcareous soil (ACS) conditions, representing a scale of differing pH values from pH 6.2 (control) up to 8.3 (severe ACS) were recorded at six time points, that are color, and shape coded in the scatter plots (14/13, 20/21, 27/28, 34/35, 41/42 and 49/50 days after sowing). (D) Manual number of side branches with PlantEye plant height max (*N* = 61 plants), 50 days after sowing. Colors and symbols indicate different growth conditions, control, and varying ACS conditions from ACS1 to ACS4 (details in Figure S2). Scatter plots were created in SPSS.

To study the accuracy of the correlation of the rosette size parameters in detail, we investigated three rosette size correlations using scatterplot analysis of the data points of the rosette diameter, 3D leaf area, manual rosette area, convex hull area, and manual rosette convex hull collected at six time points (Figure 2A–C). First, initially during the growth cycle, the strong correlation between rosette diameter and 3D leaf area (correlation coefficient 0.981) was rather square (*R* ^2^ = 0.934) than linear (*R* ^2^ = 0.887). However, starting 28 days after sowing, the scattering of the data points became stronger (Figure 2A), when flowering began (Figure S7). Second, the manual rosette area correlated very strongly with the machine 3D leaf area (correlation coefficient 0.992). This correlation was linear (*R* ^2^ = 0.964) up to 42 days after sowing (Figure 2B). Third, for the manual rosette convex hull, the strongest correlation was not found with the machine convex hull area (correlation coefficient 0.951), but with the machine 3D leaf area and the machine projected leaf area (correlation coefficient 0.976; Figure 1). This can again be explained by an effect of inflorescence stem formation on the measurements, especially beyond 41 days after sowing, as indicated by the increased scattering at those time points (Figure 2C). Hence, the correlations are best at the rosette growth stage up to the bolting stage but diminish at the advanced reproductive stages with emerged and branched inflorescence stems and siliques. ACS treatments did not affect flowering time in the two experiments (Figure S7A,B).

An interesting question was whether flowering time correlates with any PlantEye parameters and, if so, whether it can be estimated with the PlantEye. Hence, we subjected manual flowering time data collected during two experiments using the above‐described WT and *f6'h‐1* mutant data to correlation analysis with all PlantEye data collected throughout the life cycle. Notably, the three strongest negative correlations to the manual flowering time were canopy light penetration depth, plant height averaged, and plant height max 28 days after sowing (Figure S7C,D) coinciding with the average flowering time of 27 days after sowing (Figure S7). Therefore, the PlantEye measurements corresponding to a change in plant height right from the soil level reflect well the manual flowering time. As flowering time and plant height are meaningful parameters reflecting reproductive growth progression during the life cycle of *A. thaliana*, we also analyzed the plant height. Plant height averaged and the light canopy penetration depth (correlation coefficient 0.744; Figure 1) correlated equally well with the manual plant height, making both suitable to replace manual height measurements.

Next, we addressed the question of whether digital biomass was indeed the best parameter representing the plant weight in *A. thaliana* . We analyzed plants with and without inflorescence stems, imitating two growth stages. Surprisingly, while the whole plant weight indeed correlated best with the digital biomass (correlation coefficient 0.788), the rosette fresh weight correlated better with seven other PlantEye parameters (Figure 1). The digital biomass was therefore the most suitable estimator for *A. thaliana* weight only after inflorescence stems were formed, while other PlantEye parameters, like the leaf area and convex hull area, were even more suitable for non‐flowering plants.

Manual parameters related to reproductive success were the number of siliques and side branches. While the number of siliques correlated with 16 out of 20 PlantEye parameters, the number of side branches correlated with only six (Figure 1), indicating that the number of side branches was less connected to overall plant growth than the number of siliques. Those six parameters included lightness average, plant height averaged, canopy light penetration depth, plant height max, surface angle average, and the normalized difference vegetation index (NDVI) in that order of correlation strength. The correlation with the height parameters can be due to longer plants having more side branches, but the negative correlation with the lightness remains unexplained. The comparably weak correlation of the manually determined number of side branches with the PlantEye parameters makes this manual parameter less suitable for being replaced by any PlantEye parameter, highlighting the need for additional manual measurements to determine the number of side branches. A possible explanation could be that not all side branches were detected in the measurement, or the number of side branches is simply not related to overall plant growth and physiology in the tested growth conditions. To explore this further, we examined the data of the plant height max and the side branches more closely. We plotted the plant height max against the manual number of side branches obtained at 50 days, displaying the data of plants in control condition and conditions ACS1‐4 visibly distinguishable (Figure 2D). There was an apparent linear relation between plant height and the number of side branches using the ACS data but not using the control soil data, indicating an effect of the soil condition on the correlation between plant height and the number of side branches.

Strikingly, convex hull aspect ratio was the only PlantEye parameter that hardly correlated with any manual parameters (Figure 1). Therefore, when examining ACS and control soil conditions, this parameter is not suitable for estimating meaningful manual parameters in *A. thaliana* . As the convex hull aspect ratio determines the shape of the plants, it indicates that the shape of *A. thaliana* remains unchanged under the influence of ACS.

Due to the particular importance of chlorosis as a symptom in ACS, we were especially interested in PlantEye parameters useful for estimating chlorophyll content in *A. thaliana*. This aspect was examined in the rosettes of 38‐day‐old plants after their inflorescence stems were removed, by first conducting PlantEye measurements and then destructive pigment content analysis of WT and *f6'h1‐1* plants grown in four different soil conditions. The correlations of rosette leaf plant pigmentation to the PlantEye parameters were consistent among the chlorophyll *a*, chlorophyll *b*, carotenoid, and chlorophyll *a + b* content, but surprisingly not very high (Figure 1). The strongest correlation for total chlorophyll content was found with the hue, PSRI, NDVI, and lightness (correlation coefficients 0.687, −0.652, 0.579, and −0.533). There were not only significant correlations with the averages of spectral parameters, like the hue, NDVI, PSRI, and lightness, but also their bin values, further confirming their usefulness for chlorophyll estimation (Table S2). For example, the percentage of a plant's surface with a hue between 75° and 100°, a lightness between 0% and 25%, and a NDVI between 0.3 and 0.45 were having the correlation coefficients 0.633, 0.420, and −0.601 with the chlorophyll *a + b* content, offering further options for non‐destructive pigment content determination.

**FIGURE 3:**
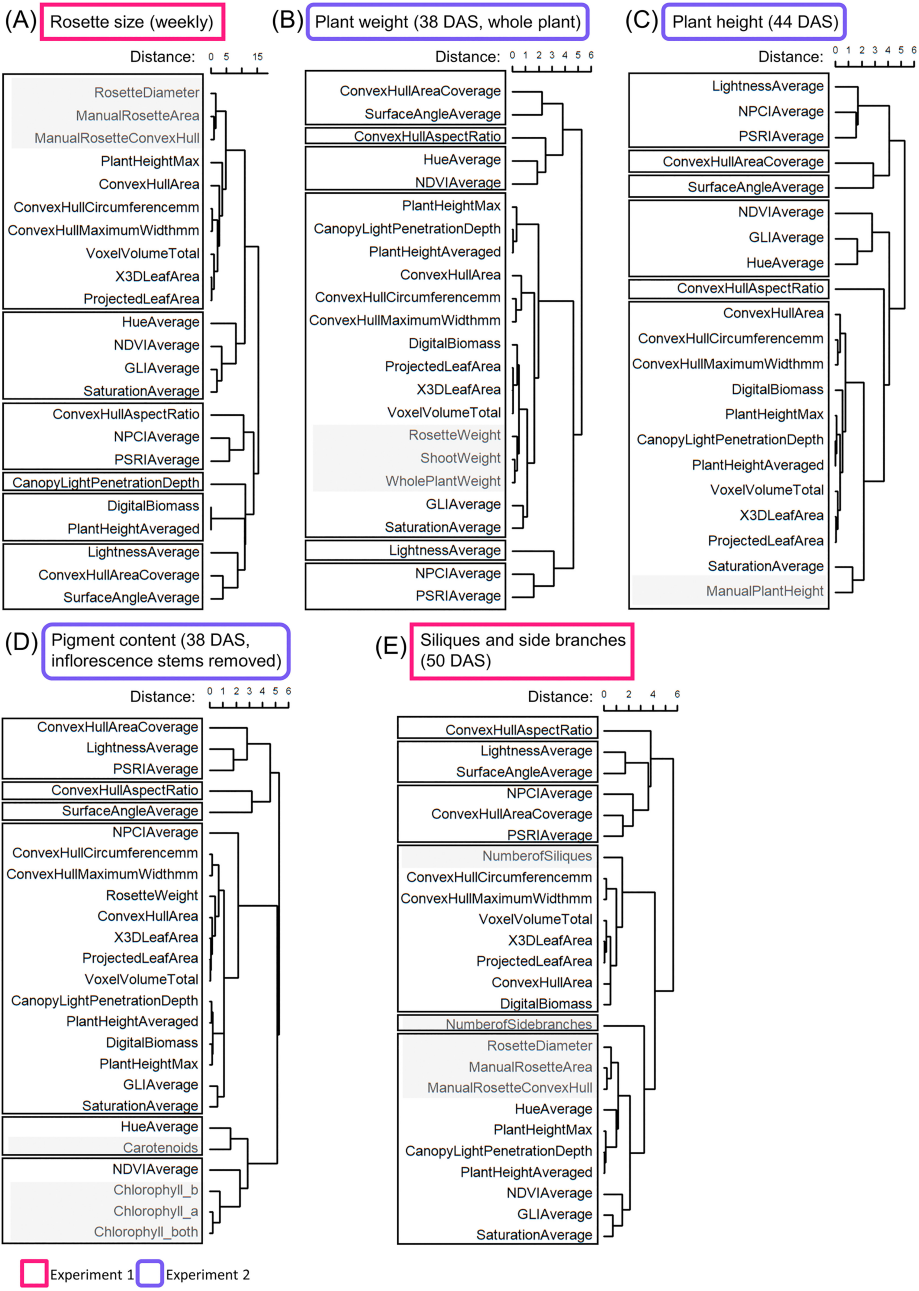
Hierarchical clustering of manual and machine‐derived phenotypic parameters. *Arabidopsis thaliana* wildtype (WT) and the coumarin‐deficient mutant *f6'h1‐1* were grown in seven alkaline calcareous soil (ACS) conditions with varying pH values in two experiments to collect manual and machine‐derived (PlantEye) data. Manual parameters were clustered with machine parameters collected at the same time points. (A) Weekly determined rosette size parameters (rosette diameter, manual rosette area, manual rosette convex hull). (B) Plant weight at 38 days after sowing (DAS; rosette weight, shoot weight and whole plant weight). (C) Manual plant height at 44 DAS. (D) Rosette pigment content at 38 DAS (chlorophyll *a*, chlorophyll *b*, chlorophyll *a + b*, carotenoids) and (E) Number of siliques and side branches at 50 DAS. Hierarchical clustering was done using the hclust function in R (stats package). Colored rectangles around boxes indicate two different experiments (Experiment 1: Pink, Experiment 2: Lavender with rounded corners). Manually determined parameters are marked with a grey box. Splitting into six clusters is highlighted by black rectangles. *N* = 23–799 data points.

Finally, we investigated the relatedness of parameters by hierarchical clustering of manual and machine phenotyping parameters. Indeed, this approach highlighted the clustering of corresponding parameters (Figure 3). For example, the manual rosette size parameters rosette diameter, manual rosette area, and manual rosette convex hull were best clustered with all corresponding morphological machine parameters such as plant height max, convex hull area, convex hull circumference, convex hull maximum width, voxel volume total, 3D leaf area, and projected leaf area (Figure 3A). Similar situations were seen for plant weight and plant height (Figure 3B,C). Remarkably, the chlorophyll *a*, *b*, and *a + b* contents clustered closest with the NDVI average and the carotenoid content with the hue average (Figure 3D). Again, the number of siliques and the number of side branches were not clustering closely together, and the number of side branches was found in a cluster separate from all machine parameters and also other manual parameters, indicating low similarity to those (Figure 3E).

In conclusion, machine‐aided phenotyping with the PlantEye was found to deliver accurate and reliable data that were meaningful for growth physiology in *A. thaliana* plants grown in ACS conditions. We identified optimal machine‐aided parameters, such as 3D leaf area, hue average, NDVI average, PSRI average, and lightness average, covering well the manually phenotyped traits. The best time point for reliable machine phenotyping of *A. thaliana* was around the onset of inflorescence stem elongation at days 34–35. Rosette size was found to be a meaningful trait for overall plant performance of *A. thaliana* in ACS, correlating with morphological and plant color traits. The number of siliques was more suited for correlation with machine parameters than the number of side branches.

### 3.2 Assessment of Machine‐Aided Phenotypic Analysis of Wildtype and the Coumarin‐Deficient Mutant *f6'h1‐1* in Seven Artificially Created ACS Conditions

Having established and validated machine phenotyping, we then inspected closely the obtained plant data at days 27–28 and 34–35, focusing on the best suited machine‐aided phenotyping parameters to assess differences in growth physiology between WT and *f6'h1‐1* plants in the ACS conditions described above and in Experiments 1 and 2 (Figures S1A, S2). Our aim was to identify one ACS condition that distinguishes the best WT and *f6'h1‐1* plants with machine phenotyping and that still allows plants to complete their life cycles. We predicted that this was the case for an intermediate ACS out of seven tested conditions (details in Figure S2B and Supporting Information). Having such an ACS condition combined with meaningful PlantEye parameters will allow us in the future to investigate genetic adaptation to ACS in this species in clearly defined soil conditions with reduced time expenses for phenotyping.

**FIGURE 4:**
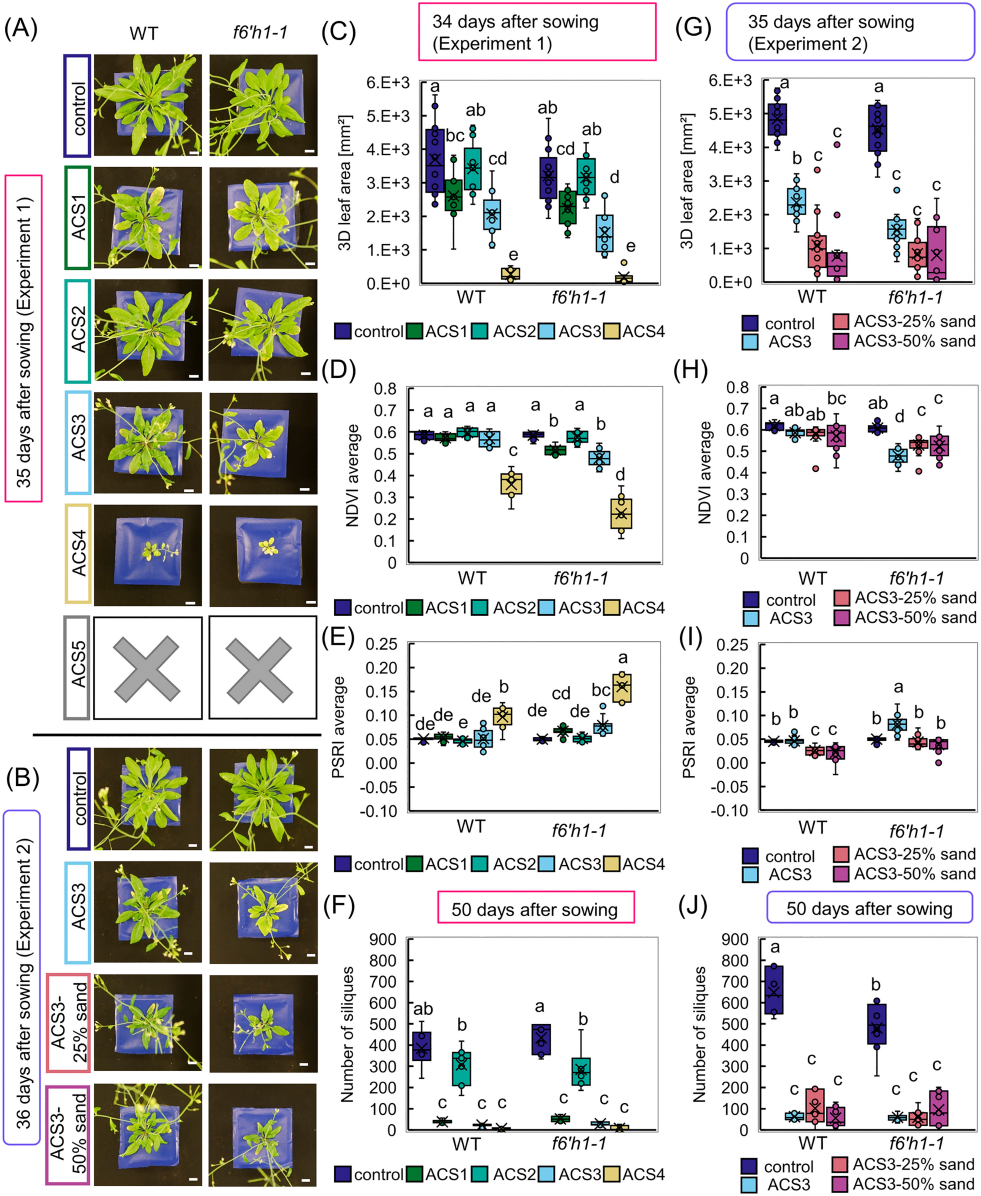
Machine‐aided phenotypic analysis of wildtype and the coumarin‐deficient mutant *f6'h1‐1* in seven artificially created ACS conditions. *Arabidopsis thaliana* wildtype (WT) and the coumarin‐deficient mutant *f6'h1‐1* were grown in seven alkaline calcareous soil (ACS) conditions with varying pH values in two experiments to determine an intermediate condition differentiating the phenotypes of lines. Selected phenotypic parameters are shown. (A, B) Photos of plants in ACS conditions. Scale bar = 1 cm. Plants in ACS5 had died at that time point. (C) 3D leaf area, (D) normalized difference vegetation index (NDVI) average, (E) plant senescence reflectance index (PSRI) average, and (F) manual number of siliques of the plants in Experiment 1 at 34 and 50 days after sowing (DAS). Conditions from left to right were control and ACS1‐5. (G) 3D leaf area, (H) NDVI average, (I) PSRI average, and (J) manual number of siliques of the plants in Experiment 2 at 35 DAS and 50 DAS. Conditions from left to right were, control, ACS3, ACS3‐25% sand, and ACS3‐50% sand. Letters indicate statistical differences (Two‐way ANOVA, Tukey test in R, *p* = 0.05, *N* = 9–16 plants).

When we displayed the plant phenotyping data (Figures 4, S8), we excluded the strong ACS5 condition because plants had died at 28 days after sowing (DAS; Figures 4A, S8A). We also excluded the mild ACS2 condition due to the lack of phenotypes differing from control (Figures 4C–E, S8C–G) except for the number of siliques in *f6'h1‐1* (Figure 4F). Reduced plant size was found at 34–35 days in both WT and the *f6'h1‐1* lines in ACS1 (Figure 4C), in ACS3 (Figure 4C,G), in ACS4 (Figure 4C), in ACS3‐25% sand (Figure 4G) and in ACS3‐50% sand (Figure 4G) It was also visible at 27–28 days in ACS3, ACS4, ACS3‐25% sand, ACS3‐50% sand (Figure 8C,H). Among the intermediate ACS conditions, leaf color changes and differences between *f6'h1‐1* and WT were found in ACS1, ACS3, ACS4, ACS3‐25% sand, and ACS3‐50% sand (Figures 4D,E,H,I, S8D–G,I–L). The number of siliques was reduced in both lines in ACS1, ACS3, ACS4, ACS3‐25% sand and ACS3‐50% sand (Figure 4F,J). Based on the strongly impaired growth in ACS4 and variation of individual plant growth in ACS3‐50% sand, we also excluded them from further analysis (Figure 4C,G). Since ACS3‐grown *f6'h1‐1* plants frequently showed more strongly affected spectral parameters than in ACS1 and ACS3‐25% sand, we finally opted for ACS3 as the best condition for detecting genetic variations in future experiments (Figures 4H,I, S8J).

In sum, the established manual and machine phenotyping system was suited to detect morphological and leaf color changes between control versus ACS and WT versus *f6'h1‐1*, with the intermediate ACS3 condition selected as suitable for detecting genetic variations. This setup provides a clearly defined pipeline for plant growth and non‐invasive phenotyping. It facilitates the detection of variations in *A. thaliana* lines with varying performances in ACS or any treatment leading to similar effects. The findings also confirm the importance of coumarins produced with the help of F6*'*H1 for adaptation to ACS conditions.

### 3.3 Validation of Manual and Machine‐Aided Phenotypic Analysis Using Known Regulatory Iron Homeostasis Mutants

In the final step, the chosen ACS3 condition and the selected phenotyping parameters were applied to validate the procedure using iron (Fe) homeostasis mutants with the additional aim of identifying potentially novel phenotypes appearing during the life cycle of *A. thaliana* plants (all phenotyping data in Table S3). The mutants were selected to have differing Fe deficiency‐induced leaf chlorosis symptoms, namely the severely chlorotic loss of function mutant *fit‐3* (Jakoby et al. 2004), the *pye‐1* mutant turning chlorotic on ACS (Long et al. 2010), and the Fe‐accumulating double mutant *btsl1 btsl2* (Hindt et al. 2017; Rodríguez‐Celma et al. 2019; Lichtblau et al. 2022). The growth phases and the influences of nutrient deficiencies on the growth cycle have not yet been completely shown for any of the three mutants in quantitative phenotyping experiments in an ACS condition. We expected that the mutants might differ in their growth curves on ACS in comparison to the wildtype, revealing novel phenotypes allowing us to resolve their functional context during the life cycle.

**FIGURE 5:**
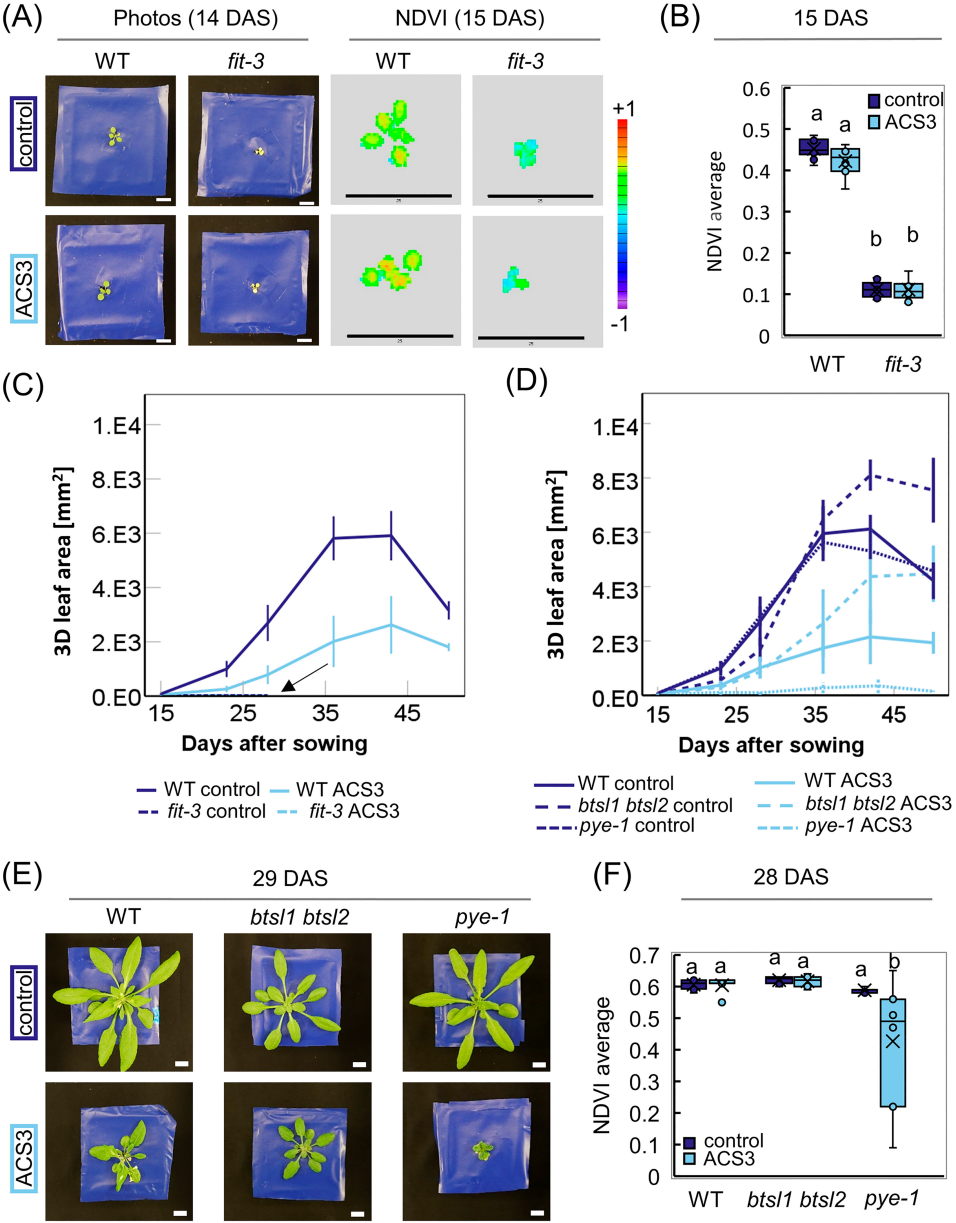
Machine‐aided phenotypic analysis using known regulatory iron homeostasis mutants. The chosen alkaline calcareous soil (ACS3) condition and the selected phenotyping parameters were applied to validate the procedure using the iron (Fe) homeostasis mutants *fit‐3, pye‐1*, and *btsl1 btsl2*. (A) Photos and normalized difference vegetation (NDVI) images (PlantEye) of wildtype (WT) and *fit‐3* mutant 14/15 days after sowing (DAS). Scale bar photos = 1 cm, scale bar NDVI images = 25 mm. Scale of the NDVI ranging from purple −1 to red +1. (B) Quantification of the NDVI of the WT and *fit‐3* at 15 DAS. *N* = 4–8 plants. (C) Development of the 3D leaf area of WT and *fit‐3* (arrow) in control soil and ACS3 soil over the experiment. *N* = 4–8 plants. (D) Development of the 3D leaf area of WT, *btsl1 btsl2*, and *pye‐1* over the time of the experiment. *N* = 4–8 plants. (E) Photos of WT, *btsl1 btsl2*, and *pye‐1* in control and ACS3 at 29 DAS. (F) NDVI of WT, *btsl1 btsl2*, and *pye‐1* at 28 DAS, *N* = 7–8 plants. Letters indicate statistical differences (Two‐way ANOVA, Tukey test in R, *p* = 0.05, *N* = 7–8 plants).

First, we analyzed the *fit‐3* mutant. It showed visibly reduced growth in control and ACS3 conditions 14 days after sowing as compared to the WT (Figure 5A). Reduced NDVI was visible in plant images (Figure 5A) and quantitatively confirmed (Figure 5B). Interestingly, in the *fit‐3* mutant, there was no increase in the 3D leaf area at all during the experiment in either condition, while there was a strong increase for the WT from 15 to 42 days after sowing in control condition and less pronounced in ACS3 (Figure 5C). The halting of rosette growth of *fit‐3* clearly shows a growth reduction caused by the inability to take up nutrients like Fe in any growth condition. Next, *pye‐1* showed a growth curve similar to the WT in control condition. Growth of *pye‐1* in ACS was much reduced compared to the WT (Figure 5D,E) and it also had a reduced NDVI average at 28 days compared with other lines and conditions (Figure 5F). Strikingly, the *btsl1 btsl2* double mutant remained slightly smaller than the WT in control and similarly large to WT in ACS3 condition at earlier growth phases, before it increased its 3D leaf area in both conditions between 28 and 35 days after sowing and then grew to an even bigger size than the WT (Figure 5D). This elevated growth performance of *btsl1 btsl2* versus WT happened at an earlier time point in ACS3 than in control (Figure 5D). The NDVI of the *btsl1 btsl2* double mutant was unchanged compared to the WT at 28 days (Figure 5F).

In conclusion, among the investigated mutants, *fit‐3* and *pye‐1* showed growth reduction and chlorosis, while the *btsl1 btsl2* mutant displayed a growth increase in ACS versus WT. These phenotypes could be detected by machine phenotyping during the plants life cycle. This validates the use of rapid, precise, and reliable machine phenotyping on ACS3 for investigating genetic adaptation of *A. thaliana* plants on ACSs. Since *fit‐3*, *pye‐1*, and *btsl1 btsl2* mutants are Fe homeostasis mutants, growth on ACS3 is influenced by their capacities to manage Fe homeostasis.

## 4 Discussion

This study established reliable and accurate procedures for growing *A. thaliana* in ACS and phenotyping plants using both manual and machine‐aided methods across their life cycle. We identified an optimal ACS condition (ACS3), enabling clear differentiation of leaf color changes and growth patterns between WT and Fe homeostasis mutants using machine‐aided phenotyping (PlantEye). Our here‐established machine phenotyping pipeline is applicable to support even small plant research labs with limited space and budget in their endeavors to uncover the molecular‐physiological and developmental phenotypes in the model species *A. thaliana* .

### 4.1 Machine‐Aided Phenotyping Across the Life Cycle of *A. thaliana* Plants Growing on ACS Is Reliable and Accurate

Reliable phenotyping requires broad phenotype testing, treatment differentiation, and ground‐truth validation (Nguyen et al. 2016; Ziamtsov and Navlakha 2019; Manavalan et al. 2021). PlantEye data effectively distinguished ACS and control plants, matching manual measurements in plant size and leaf color (Figure 4 and Figure S6). Notably, the PlantEye performed consistently across two independent experiments (Figure 4D,H), underlining reliability. 3D leaf area showed high correlation with ground‐truth manual rosette measurements, especially before inflorescence formation (Figure 2A,B). Remarkably, although chlorophyll‐spectral parameter correlations were not as strong as for rosette size, pre‐bolting and round‐bolting measurements (27/28 and 34/35 days) were already sufficient to differentiate growth conditions and genotypes based on spectral parameters (Figure 4, Figure S8). Analysis of the development of the 3D leaf area at 15–50 days and the NDVI at 15–35 days additionally indicated that judging from the growth curves, differences between lines and conditions were visible earlier in the NDVI than in the 3D leaf area (Figure S9A–E).

### 4.2 Different Machine Parameters of the PlantEye Correlated With Manually Determined Parameters

The key PlantEye parameter 3D leaf area accurately replaced manual measurements of rosette diameter and area until 42 days after sowing (Figure 2A,B). Spectral parameters—hue, NDVI, PSRI, and lightness—correlated with chlorophyll content, reducing the effort for manual assessment. Additional correlations were observed with siliques, plant weight, and flowering time (Figure 1). Rosette diameter strongly correlated with 3D leaf area (*R* ^2^ = 0.934), which also correlated with manual rosette area (*R* ^2^ = 0.964; Figure 2A,B), consistent with similar findings in crops like soybean and peanut (Vadez et al. 2015; Manavalan et al. 2021). Due to potential diurnal leaf movement (Poorter et al. 2023), 3D leaf area is more reliable than projected leaf area. Whole plant weight also correlated with digital biomass (*R* ^2^ = 0.881) after inflorescence stem formation, similar to wheat and rye studies (Bazhenov et al. 2023).

Notably, while no previous studies linked PlantEye parameters to the number of siliques and side branches, we found a correlation between the number of siliques and the plant area (e.g., convex hull area and 3D leaf area) that also correlated with the rosette diameter, suggesting a link between rosette size and reproductive fitness (Clauss and Aarssen 1994). This may be genotype‐ and environment‐dependent and requires further characterization. Side branch correlations were weak, though data hinted at condition‐dependent correlations (Figure 2D). Our study showed fewer side branches in higher control plants than in smaller ACS plants. This observation was contradictory to a study in melon plants reporting reduced shoot branching under alkaline conditions (Ulas et al. 2019). Further investigation would be needed.

Since chlorosis is a key iron deficiency symptom in ACS (Abadía et al. 2011) spectral parameters such as hue, PSRI, NDVI, and lightness are important traits for chlorophyll estimates. Despite that, the correlations to PlantEye parameters were weaker than those of rosette area measurements to respective PlantEye parameters. One reason could be that the color parameters are not optimal for single plant analysis, but rather suited for overall plant health in a field or given area (Merzlyak et al. 1999; Huang et al. 2021; Hassan and Gutub 2022). The correlation of hue, PSRI, NDVI, and lightness with chlorophyll content aligns with previous reports (Merzlyak et al. 1999; Castro and Sanchez‐Azofeifa 2008; Wu et al. 2008; Sass et al. 2012; Chen et al. 2021). Less correlating indices, like NPCI and GLI, have a high applicability in different contexts, like estimation of nutrient status (Peñuelas et al. 1994), automatic differentiation of wheat and soil for quantification of plant damage in fields (Louhaichi et al. 2001), nutritional value of plants (Andressa Alves et al. 2023), or stress in *A. thaliana* (Yang et al. 2022). For our specific purpose and technique, however, GLI was not suitable for pigment content estimation.

Flowering time correlated negatively (*R* = −0.83 to −0.84) with canopy light penetration depth and plant height just around bolting (Figure S7), because plants taller at that time point had flowered earlier. With repeated measurements around the flowering time, the PlantEye could be used to estimate flowering time based on inflorescence stem length. Other definition methods of flowering time, like first visible bud, number of leaves, or flower opening, were not tested due to the need for higher resolution for the distinction of separate, possibly overlapping, leaves. The convex hull aspect ratio was also not suitable for analyzing *A. thaliana* in ACS. It can be used for other applications like species or growth stage determination (Choudhury et al. 2016; Haque and Haque 2018).

Ultimately, NDVI, lightness, hue, and PSRI proved effective for chlorosis detection, while 3D leaf area was the best parameter for manual rosette size estimation, supporting the idea that the PlantEye is a valuable tool for machine‐aided *A. thaliana* phenotyping in ACS.

There were also limitations in phenotyping *A. thaliana* with the PlantEye. While PlantEye data correlated with reproductive traits, the system cannot directly spot siliques or side branches in 3D models of growing plants, limiting its reliability for silique counts. Moreover, alkaline conditions reduce silique size (Jain and Schmidt 2024), complicating reproductive fitness assessments. Inflorescence stem growth also introduced measurement inaccuracies. Fixing shoots in an upright position might improve precision. Nevertheless, 3D leaf area measurements remained reliable until 41/42 days to assess the rosette size and thereby estimate plant performance (Figure 2B,C). The best measurement window was at 34/35 days, around a week after bolting, when ACS and control differences were most pronounced (Figures 4, 5, and S8). After that, the data started scattering (Figure 2) and inflorescence stems overshadowed the rosette. Future studies should examine correlations between spectral parameters and chlorophyll content over time for improved assessments. Also, to assess the full versatility of the PlantEye system, it would be very interesting to compare other nutrient deficiencies and stress effects.

### 4.3 The Machine Phenotyping of WT and Fe Homeostasis Mutants Detected Expected and New Leaf Chlorosis Phenotypes on a Suited ACS Condition

The selected condition ACS3 produced a consistent size reduction of WT, one Fe deficiency and one Fe homeostasis mutant, *f6'h1‐1* and *pye‐1*, comparable to the reduced size of *A. thaliana* lines in natural ACS (Terés et al. 2019), and was consistent across three experiments (Figures 4A,B and 5E). It was shown that upon iron deficiency treatment, rosette growth can arrest (Truong et al. 2024), and this is most pronounced in young leaves (Ngigi et al. 2025). However, we did not observe a complete arrest in rosette growth of the WT, but rather a reduced growth as observed in natural ACS (Terés et al. 2019). Complete growth arrest was only the case in *fit‐3* (Figure 5C) and partly in *pye‐1* (Figure 5D). Future studies could investigate the reasons behind reduced rosette growth and especially the role of Fe availability. The difference in size between WT, *f6'h1‐1* (Figure S9A–E), *fit‐3* (Figure 5C), and *pye‐1* (Figure 5D) occurred after a visible difference was detected in the NDVI (Figure S9B–G). One likely explanation is that leaf chlorosis can be picked up rapidly by the PlantEye, while the diminished leaf growth affected the rosette size only with a delay. An alternative explanation is that leaf pigmentation and rosette size follow different dynamics.

The affected spectral parameters in *f6'h1‐1* in ACS aligned with previous reports on chlorosis in alkaline soils (Schmid et al. 2014) similar to some natural lines in natural ACS (Terés et al. 2019). The reproducibility and effect on plant growth make ACS3 a reliable tool for growing and phenotyping *A. thaliana* lines.

The *fit‐3* mutant is severely iron deficient (Jakoby et al. 2004) and failed to grow in both control and ACS3 conditions, emphasizing FIT's crucial role in iron homeostasis overriding any growth condition effect (Connorton et al. 2017; Schwarz and Bauer 2020). The FIT transcription factor is a master regulator controlling multiple downstream genes, including *F6'H1*. The downstream genes perform various reactions in Fe acquisition and metal homeostasis under Fe deficiency in the root (Schwarz et al. 2020). The *pye‐1* mutant, previously reported to arrest at the cotyledon stage in alkaline soil (Long et al. 2010), remained growth‐impaired even when transplanted to ACS3 at day eight, suggesting persistent sensitivity beyond early development. Single plants did not survive in ACS3 with death occurring between 28 and 35 days. This was well visible when NDVI average data of single plants were displayed (Figure S9G). The leaf chlorosis, seemingly more prevalent in the oldest leaves of *pye‐1* plants (Figure 5E,F), may indicate the mutants' inability to mobilize Fe or control proper levels of other heavy metals like zinc and manganese. This observation coincides with a previous report on differing roles of old and young leaves in metal ion homeostasis in *A. thaliana* (Ngigi et al. 2025). The *btsl1 btsl2* mutant (Hindt et al. 2017) had not been tested in ACS before. It initially showed reduced growth but later surpassed WT in both conditions, with an earlier growth recovery in ACS3 (Figure 5D). This is highly interesting as it might suggest a potential advantage of the *btsl1 btsl2* loss of function in alkaline conditions while maintaining normal growth in iron‐sufficient environments. That aligns with previous reports of improved growth in iron‐limited conditions without iron toxicity symptoms under iron‐sufficient conditions (Hindt et al. 2017). In agreement with a functional model proposed by Lichtblau et al. 2022, the better growth of the *btsl1 btsl2* mutant can be explained by reduced downregulation of transcription factors of the group IVc, including bHLH104, ILR3, bHLH115, and bHLH34, which promote the Fe uptake response when BTSL1 and BTSL2 proteins are not present (Hindt et al. 2017; Lichtblau et al. 2022). Overexpression of those bHLH transcription factors like bHLH104 has likely a similar effect as *btsl1 btsl2* knockout to stimulate Fe uptake (Zhang et al. 2015; Liang et al. 2017; Wang et al. 2017). Normally, the BTS (L)‐type E3 ligase proteins may bind bHLH transcription factors like FIT, PYE, ILR3, and bHLH104 and prepare them for degradation through protein ubiquitination (Selote et al. 2014; Rodríguez‐Celma et al. 2019; Spielmann et al. 2023). Clearly, the here‐presented phenotyping pipeline is suitable to detect plant phenotypes of novel Fe homeostasis mutants reliably. The study highlights the benefits of multi‐stage phenotyping to capture dynamic growth patterns. Future studies should replicate findings for *pye‐1* and *btsl1 btsl2* mutant interactions and explore additional iron‐regulatory mutants to better understand ACS tolerance mechanisms in *A. thaliana* .

A limitation of the ACS soil recipe is the possible variability of the peat composition. Using reference lines of *A. thaliana* with a known reaction across experiments can help mitigate this potential effect. Uncontrolled humidity and blue foil may have influenced the plant responses to water availability. While artificial ACS conditions offer higher controllability and consistency across labs, they do not fully reflect the natural ACS structure. Future studies could explore the comparability of artificial ACS with different natural ACS conditions to further validate the system.

In conclusion, this study outlines the preparation of several ACS conditions, with ACS3 emerging as an effective tool for detecting phenotypic differences in *A. thaliana* wildtype (Col‐0) and Fe homeostasis mutants. Importantly, in the future, this growth condition will serve as an assessment tool for growth phenotypes of mutants that help to further depict the intricacies of Fe regulatory mechanisms.

## 5 Conclusions

This study achieved multiple key objectives: (1) demonstrating an affordable, reliable, and accurate non‐invasive high‐throughput phenotyping pipeline to be applied by a single lab working with a small rosette plant species like *A. thaliana* ; (2) identifying optimal phenotyping parameters for *A. thaliana* in ACS using the PlantEye; (3) defining ACS3 as a condition that effectively differentiates wildtype from *f6'h1‐1*, and (4) identifying new growth phenotypes of Fe homeostasis mutants using this established pipeline. These findings lay a foundation for further research on plant adaptation to ACS and numerous other stress factors. The successful application of the PlantEye highlights its potential for large‐scale non‐invasive studies on plant stress responses to ACS, opening up new opportunities for future research on *A. thaliana* mutants and natural accessions. Beyond that, provided some adjustments, this procedure is easily applicable to any plant species of a comparable size that can be assessed by the MicroScan device with the PlantEye.

## Author Contributions

The conceptualization was done by P.B. and M.C.K. Methodology, investigation, data analysis, and writing of the original draft were done by M.C.K. Review and editing were carried out by all authors, supervision, and funding acquisition were done by P.B.

## Supporting information


**Table S1:** **All Phenotypic Data Collected Manually and Machine‐aided (PlantEye) for Correlation Analysis.**
Data of twelve suitable manual and 20 machine‐derived PlantEye parameters for *A. thaliana* wildtype (WT) and its coumarin‐deficient mutant *f6’h1‐1* under control and up to seven alkaline calcareous soil (ACS) conditions, representing a scale of differing pH values from pH 6.2 (control) up to 8.3 (severe ACS) were recorded during two experiments.
Manual parameters and PlantEye were determined at the indicated time points in days after sowing (DAS). Both whole plants or rosettes only were used for the determination of the parameters as indicated in the table. For details on the parameters see the material and methods section. For the PlantEye data, averages of repeated measurements per time point are shown. Block ID of plants are unique identifiers within the experiments. In case of empty fields, data were not available for the parameters at the time point for the plant.

**Table S2:** **Results of Correlation Analysis of All Manually Determined and All Machine‐derived PlantEye Parameters**.
Data of twelve suitable manual and 20 machine‐derived PlantEye parameters for *A. thaliana* wildtype (WT) and its coumarin‐deficient mutant *f6’h1‐1* under control and up to seven alkaline calcareous soil (ACS) conditions, representing a scale of differing pH values from pH 6.2 (control) up to 8.3 (severe ACS) were recorded during two experiments and subjected to correlation analysis.
Plant weight, rosette fresh weight, and pigment contents were determined 38 days after sowing. Plant height was determined 44 days after sowing. Rosette diameter, manual rosette area, and manual rosette convex hull were measured weekly during six weeks. N depended on parameters; if several time points were measured, plants were measured repeatedly. N (plant weight, rosette fresh weight, plant height) = 24, N (Rosette diameter) = 799, N (manual rosette area, manual convex hull area) = 179, N (Number of siliques and side branches) = 61, N (pigment contents) = 23. Only rosettes were used for chlorophyll content measurement in acetone. For details on parameters see materials and methods section. Spearman Rho Correlation was done in SPSS. Significant correlations are marked (* < 0.05, ** < 0.01).

**Table S3:** **All Machine‐Derived Data for Phenotypic Analysis Using Known Regulatory Iron Homeostasis Mutants.**
The ACS3 condition and the selected phenotyping parameters were applied to validate the procedure using iron (Fe) homeostasis mutants with the additional aim of identifying potentially novel phenotypes appearing during the life cycle. The iron homeostasis mutants *fit‐3, pye‐1*, and *btsl1 btsl2* were phenotyped in ACS3 weekly. Eight plants per line were planted. The data were obtained at the time points indicated in the column days after sowing. The Block ID served as a unique identifier for each plant. Note that some mutant plants did not survive.

**Data S1:** Supporting Information Containing Supplemental Figures and Supplemental Methods.

## Data Availability

All phenotypic data that support the findings of this study are available in the Supporting Information of this article.
