# Supplementary material for: Affordable Non‐Invasive Machine‐Aided Phenotyping Identifies Phenotypic Variation to Soil Stress Across the Arabidopsis thaliana Life Cycle
Source: Physiol Plant. 2025 Aug 7;177(4):e70427. doi: 10.1111/ppl.70427 (PMC12329706; doi:10.1111/ppl.70427)
Supplement: Supplementary file 2 — Table S2: Results of Correlation Analysis of All Manually Determined and All Machine‐derived PlantEye Parameters. Data of twelve suitable manual and 20 machine‐derived PlantEye parameters for A. thaliana wildtype (WT) and its coumarin‐deficient mutant f6’h1‐1 under control and up to seven alkaline calcareous soil (ACS) conditions, representing a scale of differing pH values from pH 6.2 (control) up to 8.3 (severe ACS) were recorded during two experiments and subjected to correlation analysis. Plant weight, rosette fresh weight, and pigment contents were determined 38 days after sowing. Plant height was determined 44 days after sowing. Rosette diameter, manual rosette area, and manual rosette convex hull were measured weekly during six weeks. N depended on parameters; if several time points were measured, plants were measured repeatedly. N (plant weight, rosette fresh weight, plant height) = 24, N (Rosette diameter) = 799, N (manual rosette area, manual convex hull area) = 179, N (Number of siliques and side branches) = 61, N (pigment contents) = 23. Only rosettes were used for chlorophyll content measurement in acetone. For details on parameters see materials and methods section. Spearman Rho Correlation was done in SPSS. Significant correlations are marked (* < 0.05, ** < 0.01). [file PPL-177-e70427-s003.docx]

| **Supplemental Table 2: Results of Correlation Ananlysis All Manually Determined and All Machine-derived PlantEye Parameters.**  Data of twelve suitable manual and 20 machine-derived PlantEye parameters for A. thaliana wildtype (WT) and its coumarin-deficient mutant *f6’h1-1* under control and up to seven alkaline calcareous soil (ACS) conditions, representing a scale of differing pH values from pH 6.2 (control) up to 8.3 (severe ACS) were recorded during two experiments and subjected to correlation analysis.  Plant weight, rosette fresh weight and pigment contents were determined 38 days after sowing. Plant height was determined 44 days after sowing. Rosette diameter, manual rosette area and manual rosette convex hull were measured weekly during six weeks. N depended on parameters, if several time points were measured, plants were measured repeatedly. N (plant weight, rosette fresh weight, plant height) =24, N (Rosette diameter) =799, N (manual rosette area, manual convex hull area) =179, N (Number of siliques and side branches) = 61, N (pigment contents) = 23. Only rosettes were used for chlorophyll content measurement in acetone. For details on parameters see materials and methods section. Spearman Rho Correlation was done in SPSS. Significant correlations are marked (* <0.05, ** <0.01). | Rosette diameter [cm] | Manual rosette area [mm^2^] | Manual rosette convex hull [mm^2^] | Plant fresh weight [mg] | Rosette fresh weight [mg] | Plant height [cm] | Number of siliques | Number of side branches | Chlorophyll a [µg/mg FW] | Chlorophyll b [µg/mg FW] | Carotenoids [µg/mg FW] | Chlorophyll a+b [µg/mg FW] |
| --- | --- | --- | --- | --- | --- | --- | --- | --- | --- | --- | --- | --- |
|  |  |  |  |  |  |  |  |  |  |  |  |  |
| 3D Leaf Area mm² | 0.981^**^ | 0.992^**^ | 0.976^**^ | 0.766^**^ | 0.770^**^ | 0.478^*^ | 0.818^**^ | 0.144 | -0.022 | 0.097 | -0.242 | -0.003 |
| Canopy Light Penetration Depth mm | 0.838^**^ | 0.915^**^ | 0.888^**^ | 0.698^**^ | 0.676^**^ | 0.744^**^ | 0.883^**^ | 0.435^**^ | -0.054 | 0.043 | -0.296 | -0.036 |
| Convex Hull Area Coverage % | -0.750^**^ | -0.762^**^ | -0.755^**^ | -0.083 | -0.093 | -0.646^**^ | -0.666^**^ | -0.095 | -0.529^**^ | -0.544^**^ | -0.300 | -0.522^*^ |
| Convex Hull Area mm² | 0.971^**^ | 0.969^**^ | 0.951^**^ | 0.731^**^ | 0.770^**^ | 0.608^**^ | 0.926^**^ | 0.169 | 0.021 | 0.141 | -0.218 | 0.041 |
| Convex Hull Aspect Ratio | 0.285^**^ | 0.107 | 0.144 | 0.098 | -0.174 | 0.042 | -0.250 | -0.100 | 0.249 | 0.264 | 0.278 | 0.263 |
| Convex Hull Circumference mm | 0.969^**^ | 0.966^**^ | 0.949^**^ | 0.675^**^ | 0.779^**^ | 0.616^**^ | 0.923^**^ | 0.153 | 0.033 | 0.157 | -0.210 | 0.052 |
| Convex Hull Maximum Width mm | 0.964^**^ | 0.956^**^ | 0.937^**^ | 0.630^**^ | 0.777^**^ | 0.633^**^ | 0.913^**^ | 0.150 | 0.025 | 0.136 | -0.211 | 0.040 |
| Digital Biomass mm³ | 0.897^**^ | 0.973^**^ | 0.951^**^ | 0.788^**^ | 0.751^**^ | 0.519^**^ | 0.906^**^ | 0.231 | -0.056 | 0.060 | -0.286 | -0.036 |
| GLI Average | 0.712^**^ | 0.552^**^ | 0.591^**^ | 0.691^**^ | 0.750^**^ | 0.229 | 0.575^**^ | 0.228 | 0.001 | 0.072 | -0.277 | 0.015 |
| GLI [-0.2:0] % | -0.430^**^ | -0.274^**^ | -0.319^**^ | -0.339 | -0.605^**^ | -0.535^**^ | -0.772^**^ | -0.126 | -0.059 | -0.098 | 0.244 | -0.075 |
| GLI [-1:-0.2] % | 0.074^*^ | 0.042 | 0.018 |  |  | -0.256 | -0.175 | -0.075 |  |  |  |  |
| GLI [0.15:0.3] % | -0.463^**^ | -0.389^**^ | -0.449^**^ | -0.531^**^ | -0.639^**^ | -0.540^**^ | 0.468^**^ | 0.494^**^ | 0.257 | 0.164 | 0.520^*^ | 0.238 |
| GLI [0.3:0.6] % | 0.691^**^ | 0.556^**^ | 0.602^**^ | 0.688^**^ | 0.728^**^ | 0.306 | 0.536^**^ | 0.215 | -0.091 | -0.010 | -0.332 | -0.079 |
| GLI [0.6:1] % | 0.654^**^ | 0.613^**^ | 0.574^**^ | 0.665^**^ | 0.671^**^ | 0.365 | 0.318^*^ | 0.381^**^ | 0.238 | 0.302 | -0.027 | 0.249 |
| GLI [0:0.15] % | -0.705^**^ | -0.556^**^ | -0.587^**^ | -0.661^**^ | -0.763^**^ | 0.073 | -0.310^*^ | -0.112 | -0.031 | -0.105 | 0.205 | -0.041 |
| Hue Average ° | -0.259^**^ | -0.325^**^ | -0.297^**^ | -0.353 | -0.210 | 0.495^*^ | 0.841^**^ | 0.141 | 0.695^**^ | 0.578^**^ | 0.603^**^ | 0.687^**^ |
| Hue [0:25] % | -0.433^**^ | -0.296^**^ | -0.338^**^ | -0.274 | -0.581^**^ | -0.511^*^ | -0.786^**^ | -0.138 | -0.067 | -0.106 | 0.233 | -0.085 |
| Hue [100:125] % | 0.635^**^ | 0.481^**^ | 0.520^**^ | -0.041 | 0.263 | -0.113 | 0.525^**^ | 0.368^**^ | 0.557^**^ | 0.478^*^ | 0.343 | 0.547^**^ |
| Hue [125:360] % | -0.517^**^ | -0.411^**^ | -0.407^**^ | -0.610^**^ | -0.643^**^ | 0.508^*^ | 0.897^**^ | 0.196 | 0.351 | 0.244 | 0.503^*^ | 0.340 |
| Hue [25:50] % | -0.299^**^ | -0.089 | -0.146 | -0.476^*^ | -0.550^**^ | -0.637^**^ | -0.728^**^ | -0.147 | -0.119 | -0.141 | 0.124 | -0.130 |
| Hue [50:75] % | -0.297^**^ | -0.133 | -0.196^**^ | -0.463^*^ | -0.561^**^ | -0.588^**^ | -0.377^**^ | 0.259^*^ | -0.449^*^ | -0.444^*^ | -0.108 | -0.456^*^ |
| Hue [75:100] % | -0.268^**^ | -0.240^**^ | -0.244^**^ | 0.500^*^ | 0.445^*^ | 0.270 | 0.510^**^ | 0.268^*^ | -0.657^**^ | -0.533^**^ | -0.764^**^ | -0.633^**^ |
| Lightness Average % | -0.641^**^ | -0.698^**^ | -0.706^**^ | -0.458^*^ | -0.589^**^ | 0.045 | -0.206 | -0.526^**^ | -0.544^**^ | -0.529^**^ | -0.343 | -0.533^**^ |
| Lightness [0:0] % |  |  |  |  |  |  |  |  |  |  |  |  |
| Lightness [0:25] % | 0.448^**^ | 0.399^**^ | 0.430^**^ | 0.283 | 0.503^*^ | -0.487^*^ | 0.186 | 0.347^**^ | 0.409 | 0.440^*^ | 0.171 | 0.420^*^ |
| Lightness [100:100] % |  |  |  |  |  |  |  |  |  |  |  |  |
| Lightness [25:50] % | -0.441^**^ | -0.395^**^ | -0.426^**^ | -0.286 | -0.503^*^ | 0.487^*^ | -0.186 | -0.347^**^ | -0.409 | -0.440^*^ | -0.171 | -0.420^*^ |
| Lightness [50:75] % | -0.004 | -0.051 | -0.049 | 0.351 | 0.259 | 0.226 | 0.249 | -0.272^*^ | -0.269 | -0.236 | -0.103 | -0.281 |
| Lightness [75:100] % | 0.017 | 0.065 | 0.100 |  |  |  | 0.246 | 0.113 |  |  |  |  |
| NDVI Average | 0.675^**^ | 0.548^**^ | 0.579^**^ | 0.244 | 0.552^**^ | -0.344 | 0.248 | 0.326^*^ | 0.586^**^ | 0.570^**^ | 0.321 | 0.579^**^ |
| NDVI [-1:0] % | -0.309^**^ | -0.280^**^ | -0.307^**^ | -0.048 | -0.101 | -0.051 | -0.421^**^ | -0.178 | -0.458^*^ | -0.411 | -0.325 | -0.460^*^ |
|  | Rosette diameter [cm] | Manual rosette area [mm^2^] | Manual rosette convex hull [mm^2^] | Plant fresh weight [mg] | Rosette fresh weight [mg] | Plant height [cm] | Number of siliques | Number of side branches | Chlorophyll a [µg/mg FW] | Chlorophyll b [µg/mg FW] | Carotenoids [µg/mg FW] | Chlorophyll a+b [µg/mg FW] |
| NDVI [0.15:0.3] % | -0.390^**^ | -0.214^**^ | -0.264^**^ | -0.155 | -0.543^**^ | 0.756^**^ | 0.538^**^ | 0.144 | -0.504^*^ | -0.490^*^ | -0.245 | -0.502^*^ |
| NDVI [0.3:0.45] % | -0.663^**^ | -0.559^**^ | -0.586^**^ | -0.263 | -0.411^*^ | 0.396 | 0.525^**^ | 0.215 | -0.614^**^ | -0.585^**^ | -0.425^*^ | -0.601^**^ |
| NDVI [0.45:0.6] % | 0.002 | 0.033 | 0.067 | -0.360 | -0.513^*^ | -0.156 | 0.351^**^ | 0.194 | -0.450^*^ | -0.417^*^ | -0.247 | -0.430^*^ |
| NDVI [0.6:1] % | 0.823^**^ | 0.792^**^ | 0.800^**^ | 0.326 | 0.565^**^ | -0.185 | 0.133 | 0.492^**^ | 0.598^**^ | 0.573^**^ | 0.356 | 0.587^**^ |
| NDVI [0:0.15] % | -0.326^**^ | -0.170^*^ | -0.230^**^ | -0.325 | -0.570^**^ | 0.119 | -0.452^**^ | -0.138 | -0.381 | -0.401 | -0.107 | -0.384 |
| NPCI Average | 0.539^**^ | 0.494^**^ | 0.483^**^ | 0.662^**^ | 0.677^**^ | -0.136 | -0.664^**^ | 0.027 | -0.444^*^ | -0.318 | -0.708^**^ | -0.419^*^ |
| NPCI [-0.2:0] % | -0.519^**^ | -0.407^**^ | -0.410^**^ | -0.678^**^ | -0.688^**^ | 0.515^*^ | 0.891^**^ | 0.194 | 0.353 | 0.251 | 0.533^**^ | 0.336 |
| NPCI [-1:-0.2] % | 0.326^**^ | 0.339^**^ | 0.350^**^ | -0.070 | -0.013 | -0.197 | 0.797^**^ | 0.180 | 0.337 | 0.216 | 0.389 | 0.324 |
| NPCI [0.2:0.4] % | 0.423^**^ | 0.471^**^ | 0.464^**^ | 0.631^**^ | 0.631^**^ | 0.135 | -0.234 | -0.122 | -0.520^*^ | -0.398 | -0.744^**^ | -0.496^*^ |
| NPCI [0.4:0.6] % | 0.681^**^ | 0.744^**^ | 0.699^**^ | 0.622^**^ | 0.719^**^ | -0.030 | -0.052 | 0.313^*^ | 0.014 | 0.140 | -0.164 | 0.035 |
| NPCI [0.6:1] % | 0.676^**^ | 0.704^**^ | 0.654^**^ | 0.686^**^ | 0.474^*^ | 0.556^**^ | 0.271^*^ | 0.539^**^ | 0.192 | 0.221 | -0.249 | 0.211 |
| NPCI [0:0.2] % | 0.138^**^ | -0.007 | 0.012 | -0.567^**^ | -0.530^**^ | -0.468^*^ | -0.618^**^ | 0.067 | 0.412 | 0.296 | 0.647^**^ | 0.386 |
| PSRI Average | 0.251^**^ | 0.298^**^ | 0.271^**^ | 0.457^*^ | 0.422^*^ | -0.359 | -0.796^**^ | -0.178 | -0.673^**^ | -0.532^**^ | -0.715^**^ | -0.652^**^ |
| PSRI [-0.2:0] % | -0.494^**^ | -0.388^**^ | -0.391^**^ | -0.648^**^ | -0.621^**^ | 0.462^*^ | 0.874^**^ | 0.192 | 0.418^*^ | 0.300 | 0.588^**^ | 0.396 |
| PSRI [-0.4:-0.2] % | -0.440^**^ | -0.347^**^ | -0.338^**^ | -0.043 | -0.129 | 0.720^**^ | 0.895^**^ | 0.185 | -0.141 | -0.210 | -0.103 | -0.132 |
| PSRI [-0.8:-0.4] % | 0.366^**^ | 0.382^**^ | 0.388^**^ | 0.316 | 0.139 | 0.294 | 0.885^**^ | 0.155 | -0.146 | -0.007 | -0.150 | -0.113 |
| PSRI [-4:-0.8] % | 0.137^**^ | 0.146 | 0.140 |  |  |  | 0.436^**^ | -0.016 |  |  |  |  |
| PSRI [0.2:4] % | 0.025 | 0.160^*^ | 0.103 | -0.356 | -0.460^*^ | -0.364 | -0.637^**^ | -0.188 | -0.452^*^ | -0.436^*^ | -0.144 | -0.453^*^ |
| PSRI [0:0.2] % | 0.632^**^ | 0.473^**^ | 0.514^**^ | 0.583^**^ | 0.618^**^ | -0.092 | 0.021 | 0.313^*^ | -0.049 | 0.048 | -0.329 | -0.029 |
| Plant Height Averaged mm | 0.846^**^ | 0.913^**^ | 0.888^**^ | 0.693^**^ | 0.708^**^ | 0.744^**^ | 0.883^**^ | 0.439^**^ | -0.102 | 0.018 | -0.367 | -0.080 |
| Plant Height Max mm | 0.910^**^ | 0.917^**^ | 0.892^**^ | 0.674^**^ | 0.703^**^ | 0.743^**^ | 0.889^**^ | 0.407^**^ | -0.081 | 0.014 | -0.337 | -0.060 |
| Projected Leaf Area mm² | 0.980^**^ | 0.991^**^ | 0.976^**^ | 0.768^**^ | 0.770^**^ | 0.477^*^ | 0.819^**^ | 0.144 | -0.015 | 0.107 | -0.234 | 0.004 |
| Saturation Average % | 0.758^**^ | 0.643^**^ | 0.672^**^ | 0.748^**^ | 0.789^**^ | 0.075 | 0.392^**^ | 0.178 | -0.212 | -0.103 | -0.492^*^ | -0.190 |
| Saturation [0:0] % |  |  |  |  |  |  |  |  |  |  |  |  |
| Saturation [0:25] % | -0.733^**^ | -0.606^**^ | -0.634^**^ | -0.783^**^ | -0.810^**^ | 0.306 | -0.316^*^ | -0.205 | 0.193 | 0.091 | 0.428^*^ | 0.177 |
| Saturation [100:100] % | 0.215^**^ | 0.245^**^ | 0.238^**^ |  |  | 0.413^*^ | 0.361^**^ | 0.382^**^ |  |  |  |  |
| Saturation [25:50] % | 0.498^**^ | 0.385^**^ | 0.414^**^ | -0.139 | -0.090 | -0.752^**^ | 0.197 | 0.276^*^ | -0.435^*^ | -0.439^*^ | -0.089 | -0.450^*^ |
| Saturation [50:75] % | 0.800^**^ | 0.770^**^ | 0.786^**^ | 0.692^**^ | 0.731^**^ | 0.574^**^ | 0.475^**^ | 0.198 | -0.241 | -0.140 | -0.535^**^ | -0.218 |
| Saturation [75:100] % | 0.702^**^ | 0.742^**^ | 0.695^**^ | 0.596^**^ | 0.738^**^ | 0.466^*^ | 0.471^**^ | 0.522^**^ | 0.193 | 0.239 | -0.158 | 0.205 |
| Surface Angle Average ° | -0.587^**^ | -0.672^**^ | -0.636^**^ | -0.129 | -0.491^*^ | -0.265 | 0.011 | -0.394^**^ | 0.053 | 0.019 | 0.372 | 0.044 |
| Voxel Volume Total mm³ | 0.980^**^ | 0.990^**^ | 0.974^**^ | 0.764^**^ | 0.770^**^ | 0.486^*^ | 0.877^**^ | 0.164 | -0.016 | 0.107 | -0.238 | 0.003 |
| ** Correlation is significant at the 0.01 level (2-tailed). | | | |  |  |  |  |  |  |  |  |  |
| * Correlation is significant at the 0.05 level (2-tailed). | | | |  |  |  |  |  |  |  |  |  |
