# Supplementary material for: Affordable Non‐Invasive Machine‐Aided Phenotyping Identifies Phenotypic Variation to Soil Stress Across the Arabidopsis thaliana Life Cycle
Source: Physiol Plant. 2025 Aug 7;177(4):e70427. doi: 10.1111/ppl.70427 (PMC12329706; doi:10.1111/ppl.70427)
Supplement: Supplementary file 4 — Data S1: Supporting Information Containing Supplemental Figures and Supplemental Methods. [file PPL-177-e70427-s004.pdf]

Supporting Information

Affordable non-invasive machine-aided phenotyping identifies phenotypic variation to soil stress across the *Arabidopsis thaliana* life cycle

Marie Christin Knopf[1], Petra Bauer[1,2]*
[1]Institute of Botany, Heinrich-Heine-University, 40225 Düsseldorf, Germany
[2]Cluster of Excellence on Plant Science (CEPLAS), Heinrich-Heine-University, 40225 Düsseldorf, Germany

*Author for correspondence: petra.bauer@hhu.de

Content
- Supplemental Figures……………………………………………………pp. 2-19
- Supplemental Tables…………………………………...…………pp. 20-24
- Supplemental Method………………… …………………………..p. 25-27

Supplemental Figure 1

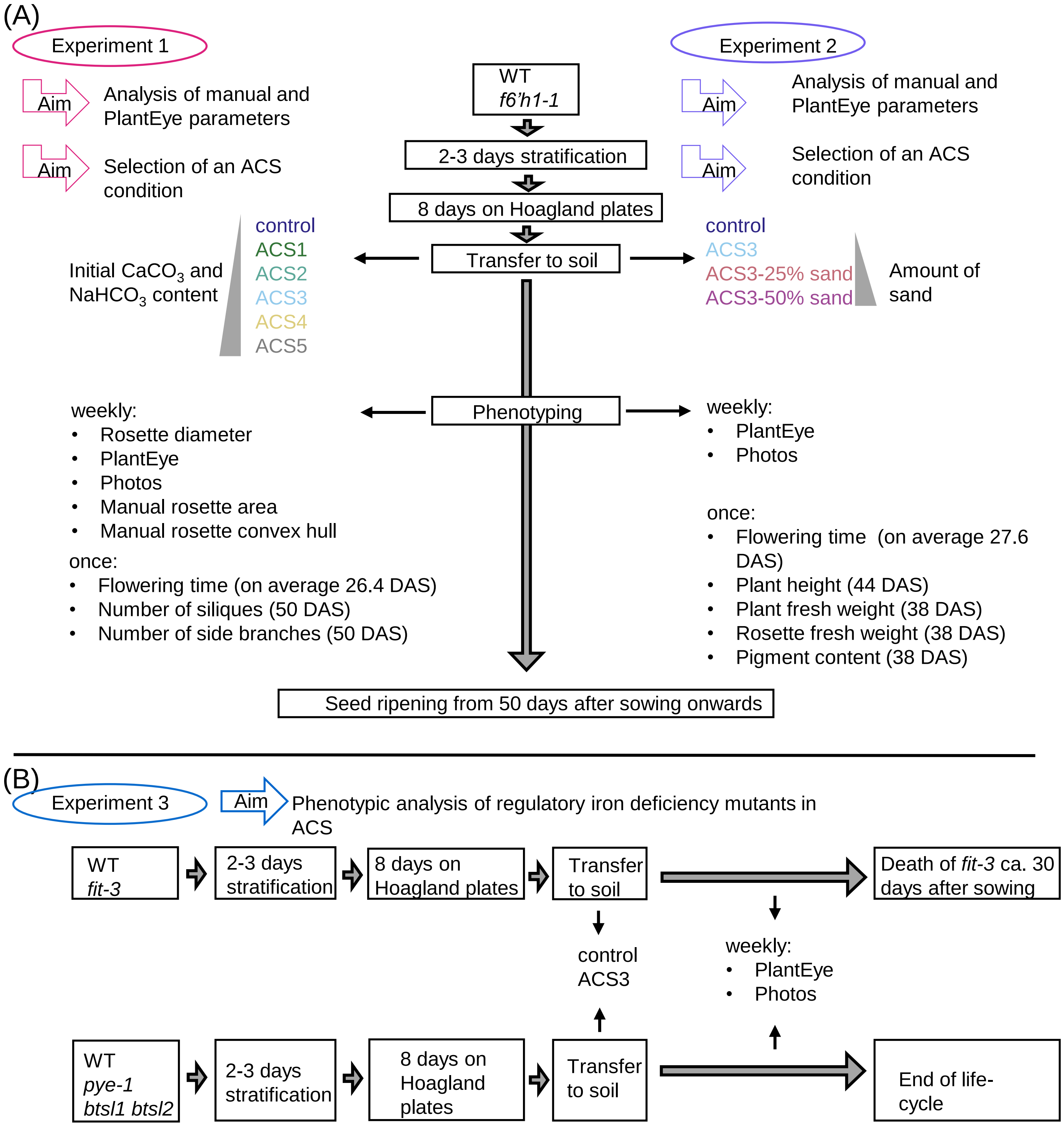

Supplemental Figure 1: Overview Over Three Experiments for Phenotypic Data Collection and Mutant Analysis.

A: The wild type (WT) and coumarin deficient mutant *f6'h1-1* were grown in two experiments (Experiment 1 and 2) in mild to severe alkaline calcareous soil (ACS) conditions to collect manual and machine-derived (PlantEye) phenotypic parameters and determine an intermediate alkaline calcareous soil (ACS) condition showing differences between the WT and *f6'h1-1***.** ACS conditions were created by adding $CaCO_3$, $NaHCO_3$ and sand to a peat-based soil substrate resulting in a pH range between 6.2 and 8.3. Phenotypic measurements were conducted throughout the plants' life cycle. **B:** In experiment 3, wildtype (WT) and three regulatory iron homeostasis mutants (*fit-3, pye-1, btsl1btsl2*) were grown in control and ACS3 and machine-derived parameters were determined to detect potential novel phenotypes.

Supplemental Figure **2**

(A)

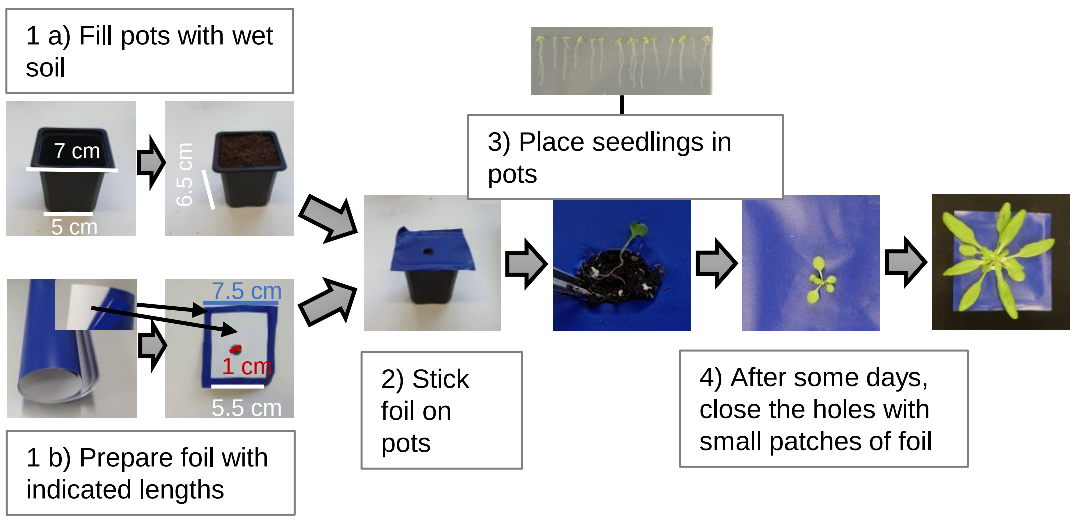

(B)

Condition	$CaCO_3$ (g/l dry soil[a])	$NaHCO_3$ (g/l dry soil[a])	Sand[b] (% wet soil)	pH[c]	Watering	Control "Top soil"[d]	Experiment 1	Experiment 2	Experiment 3
control	0	0	0	6.2 +/- 0.14	dH_2O	no	■	■	■
ACS1	6	3	0	7.6	$NaHCO_3$ solution	no	■		
ACS2	8	4	0	7.8	dH_2O	yes	■		
ACS3	8	4	0	7.85 +/- 0.13	$NaHCO_3$ solution	no	■	■	■
ACS4	31.6	13.68	0	8.3	dH_2O	yes	■		
ACS5	30	21	0	8.2	dH_2O	yes	■		
ACS3-25% sand	8	4	25	8.0	dH_2O	no		■	
ACS3-50% sand	8	4	50	8.2	dH_2O	no		■	

a Floraton 1 (Floragard), baked at 80 °C over night
b Quartz sand (Probau), washed three times with deionised water (dH_2O)
c The pH was determined in deionised water. In case a soil condition was prepared more than once the mean +/- standard deviation is shown.
d All pots were filled with the same amount of soil. In some cases top soil containing control soil (20-30 g) was layered on top of pot soil

Supplemental Figure 2: Method of Planting and Preparation of Alkaline Calcareous Soil (ACS).

A: To facilitate background removal for the machine-aided measurements, soil was covered with a blue foil, before planting, leaving a hole into which eight-day-old seedlings were planted. Holes were then closed around the seedling. **B:** Detailed composition of control and different alkaline calcareous soil (ACS) conditions. Soil was prepared with a peat substrate supplemented with different amounts of $CaCO_3$, $NaHCO_3$, and sand, named control, ACS1-5, and ACS3-25% and -50% sand. The pH of the ACS conditions ranged from mild (pH 7.6) to severe (pH 8.3)

Supplemental Figure **3**

MANUAL PARAMETERS

Parameter [unit]	Description	Illustration
Rosette diameter [cm]	Average of two to three longest possible diameters per rosette. Two and three weeks after sowing it was measured using ImageJ, older plants were measured using a ruler.	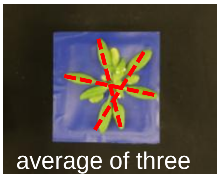
Manual rosette area [mm^2]	Area within a line drawn closely around the rosette. It was measured in ImageJ.	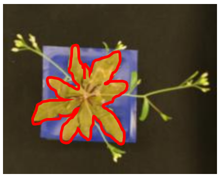
Manual rosette convex hull [mm^2]	Area of the smallest convex polygon that can be drawn around the rosette. It was measured in ImageJ.	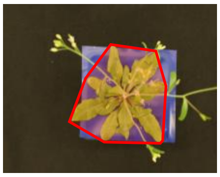
Plant fresh weight [mg]	Weight of rosette and shoots after removal of roots	
Rosette fresh weight [mg]	Weight of rosette only without shoots and roots	
Plant height [cm]	Length from the blue foil to the apical meristem of the main shoot. It was measured using a ruler.	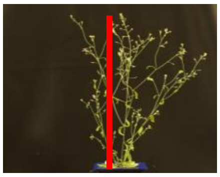
Number of siliques	Siliques were counted if they had extended further than the petals (red arrows).	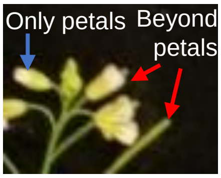
Number of side branches	Side branches were counted once they had formed a visible leaf (read arrow).	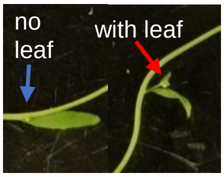
Flowering time [days after sowing]	The flowering time was the number of days after sowing at which the inflorescences was at least 1.5 cm long. It was determined with a ruler.	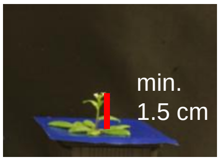

Supplemental Figure 3: **Manually Measured Phenotypical Parameters**.
The phenotypical parameters were either determined using photos and ImageJ or directly at the plants using a ruler. The rosette diameter, rosette area and rosette convex hull were determined weekly, the plant weight 38 days after sowing (DAS), the plant height at 44 DAS and the number of siliques and side branches at 50 DAS.

Supplemental Figure **4**

PLANTEYE PARAMETERS - MORPHOLOGICAL

Parameter [unit]	Type[a]	Description	Illustration
3D leaf area [mm²]	D	Plant area under consideration of the three-dimensional shape.	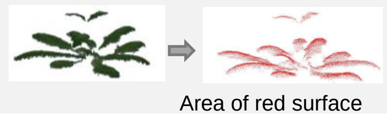
Canopy light penetration depth [mm]	D	Depth how deep laser reaches into the canopy. Depended on the density of the canopy.	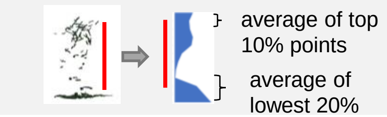
Convex hull area [mm²]	T	Area of the smallest possible convex polygon drawn around the plant.	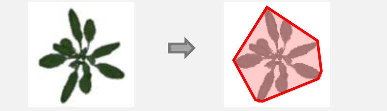
Convex hull area coverage [%]	T	Percentage of the Convex Hull overlaid by the plant.	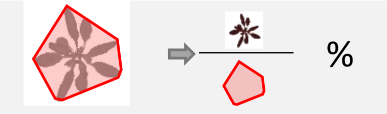
Convex hull maximum width [mm]	T	Longest possible straight line in the Convex Hull.	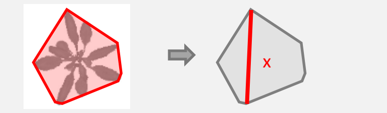
Convex hull aspect ratio [%]	T	Quotient of the longest length within the Convex Hull and the length of the perpendicular width to it at its midpoint.	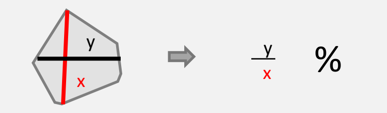
Convex hull circumference [mm]	T	Circumference of the smallest possible convex polygon drawn around the plant.	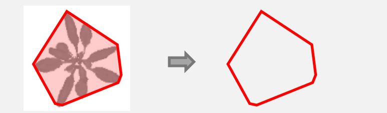
Plant height max [mm]	D	Height of the plant from the pot height to the highest point of the plant.	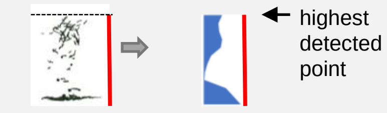
Plant height averaged [mm]	D	Average height of the highest 10% of points representing the plant.	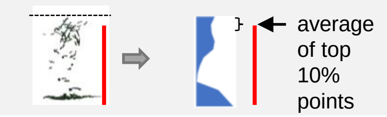
Projected leaf area [mm²]	D	Tow dimensional area covered by the plant, when seen from top view	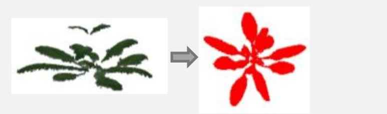
Digital biomass [mm³]	D	Product of multiplication of the 3D Leaf Area and Plant Height averaged	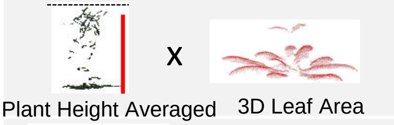
Surface angle average [°]	T	Mean angle of triangles representing the plant shape.	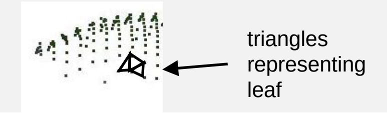
Voxel volume total [mm³]	T	The total volume of all voxels (three dimensional pixels) forming the representation of the plant	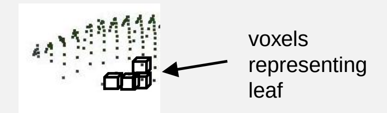

[a] Type of parameter, D= Digital Plant Parameter T= Technical Parameter

Supplemental Figure 4: Morphological Machine-Derived Parameters Measured with the PlantEye.

Thirteen different morphological parameters were measured at six time points across the life cycle. Unless indicated differently, the information on the parameters were obtained from the Phenospex-website and the manual provided by the company. The morphological parameters rely on the laser reflectance of an object. Digital Plant Parameters (here Type D) and Technical Parameters (here Type T) can be distinguished. Phena version Phena v2.0 and HortControl version 3.8.5 were used.

Supplemental Figure 5

PLANTEYE PARAMETERS - SPECTRAL

Parameter [unit]	Type[a]	Description	Illustration / Formula
Hue [°]	T	Hue is the first variable the HSL (Hue-Saturation-Lightness) colour space and ranges from 0° to 360° in a colour circle (Hassan and Gutub 2022).<br>The average per plant and the percentage of voxels within different (definable) ranges are calculated.	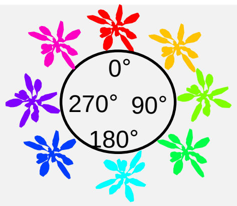
Saturation [%]	T	Saturation it the second variable in the HSL colour space ranging from grey (0%) to pure colour without grey (100%) (Hassan and Gutub 2022).<br>The average per plant and the percentage of voxels within different (definable) ranges are calculated.	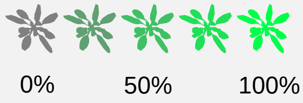
Lightness [%]	T	Lightness is the third variable in the HSL colour space, defining the brightness of the colour. It ranges from black (0%) to white (100%)(Hassan and Gutub 2022).<br>The average per plant and the percentage of voxels within different (definable) ranges are calculated.	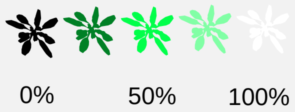
GLI	D	The Greenness Leaf Index is used to compare the reflection of green relative to that of the other colours.<br>The average per plant and the percentage of voxels within different (definable) ranges are calculated.	$\frac{2*GREEN-RED-BLUE}{2*GREEN+RED+BLUE}$
NDVI	D	The Normalized Difference Vegetation Index can be used to estimate vegetation stress, discriminate between different vegetation forms and estimate chlorophyll concentration and plant productivity (Huang et al. 2021).<br>The average per plant and the percentage of voxels within different (definable) ranges are calculated.	$\frac{NIR-RED}{NIR+RED}$
NPCI	D	The Normalized Pigment Chlorophyll Index can be used to assess chlorophyll content at late stages of plant growth (Hatfield and Prueger 2010).<br>The average per plant and the percentage of voxels within different (definable) ranges are calculated.	$\frac{RED-BLUE}{RED+BLUE}$
PSRI	D	The Plant Senescence Reflectance Index can be used to detect leaf senescence as well as fruit ripening (Merzlyak et al. 1999).<br>The average per plant and the percentage of voxels within different (definable) ranges are calculated.	$\frac{RED-BLUE}{NIR}$

[a] Type of parameter, D= Digital Plant Parameter T= Technical Parameter

Supplemental Figure 5: Spectral Machine-Derived Parameters Measured with the PlantEye. Unless indicated differently, the information on the parameters were obtained from the Phenospex-website and the manual provided by the company. Seven different spectral parameters can be determined. The colors in capital letters indicate the reflections of RED (624-634 nm), GREEN (530-540 nm), BLUE (465-485 nm) and NEAR-INFRARED (720-750 nm). For each parameter the average over the whole plant and the percentage of voxels within ranges (bins) is calculated. Digital Plant Parameters (here Type D) and Technical Parameters (here Type T) can be distinguished. Phena version Phena v2.0 and HortControl version 3.8.5 were used.

Supplemental Figure **6**

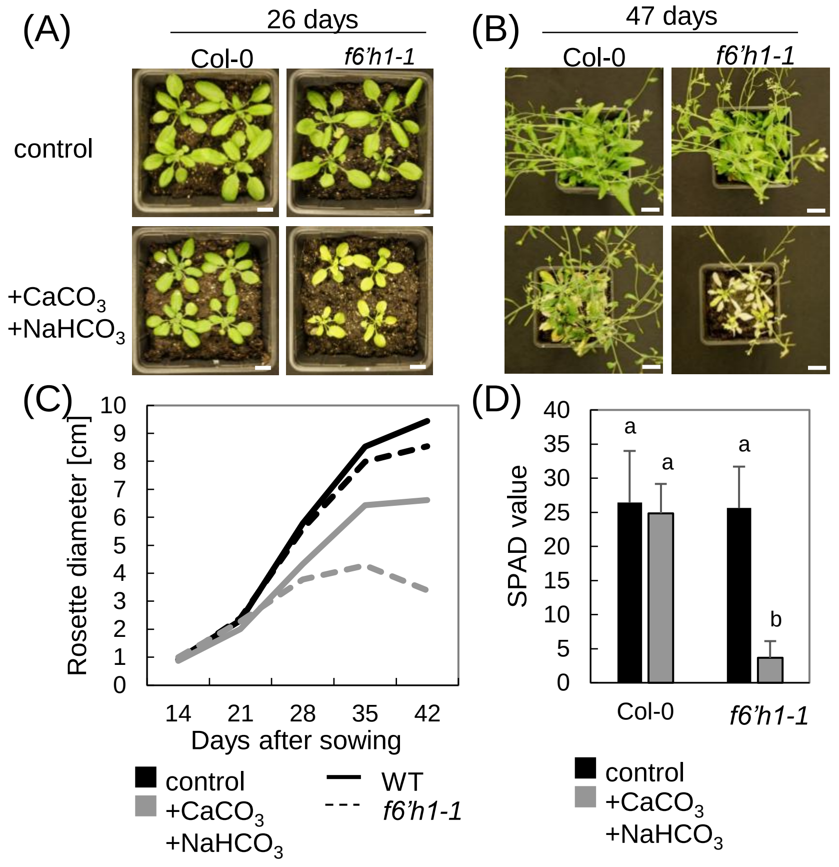

Supplemental Figure 6: Preliminary Experiment Relying on Manual Phenotyping to Distinguish Wild Type and *f6'h1-1* Mutants.

In a preliminary experiment, we confirmed that indeed growth in an alkaline calcareous soil (ACS) condition causes reduced size of WT and *f6'h1-1* with more intense visible leaf chlorosis in *f6'h1-1*, confirmed by manual measurements of rosette diameter and SPAD values. **A, B:** Photos of Col-0 and *f6'h1-1* plants in control soil and soil with $CaCO_3$ and $NaHCO_3$ added 26 and 47 days after sowing. Scale bar=1 cm. C: Development of the rosette diameter in that condition. N= 16 plants. **D:** SPAD values of Col-0 and *f6'h1-1* plants in control soil and soil with $CaCO_3$ and $NaHCO_3$ added 47/48 days after sowing. Labels indicate significantly different groups (p=0.05, Two-way ANOVA and Tukey Test in R, N=16 plants).

Supplemental Figure 7

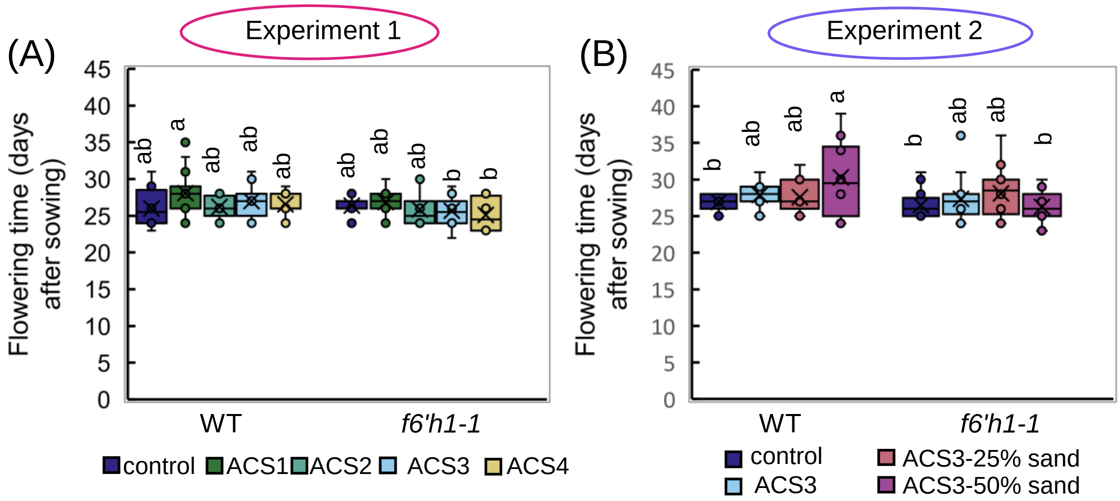

(C)

Parameters (days after sowing)	N (number of plants)	Flowering time
Canopy Light Penetration Depth (28)	264	-0.843**
Plant Height Averaged (28)	264	-0.832**
Plant Height Max (28)	264	-0.831**
GLI [0:0.15] % (49)	114	-0.481**
NDVI [0.6:1] % (49)	114	0.555**

* Correlation is significant at the 0.05 level (2-tailed).
**Correlation is significant at the 0.01 level (2-tailed).

(D)

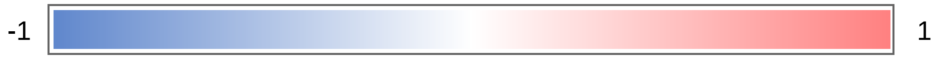

Supplemental Figure 7: **Flowering Time of Wild Type and *f6'h1-1* Mutant in Different Alkaline Calcareous (ACS) Conditions and Correlation with Machine-Derived Parameters (PlantEye).**

A: Flowering time of wildtype (WT) and *f6'h1-1* in from left to right control condition and conditions ACS1-4 (Experiment 1). **B:** Flowering time of WT and *f6'h1-1* in from left to right control condition and conditions ACS3, ACS3-25% sand and ACS3-50% sand (Experiment 2). Labels indicate statistical groups. Two-way ANOVA and Tukey test in R, p=0.05, N=8-16 plants **C**: Machine-derived parameters correlating strongest (Spearman Rho correlation coefficient) with the flowering time. **D:** Color scale of heat map (-1 blue, 0 white, +1 red).

Supplemental Figure 8

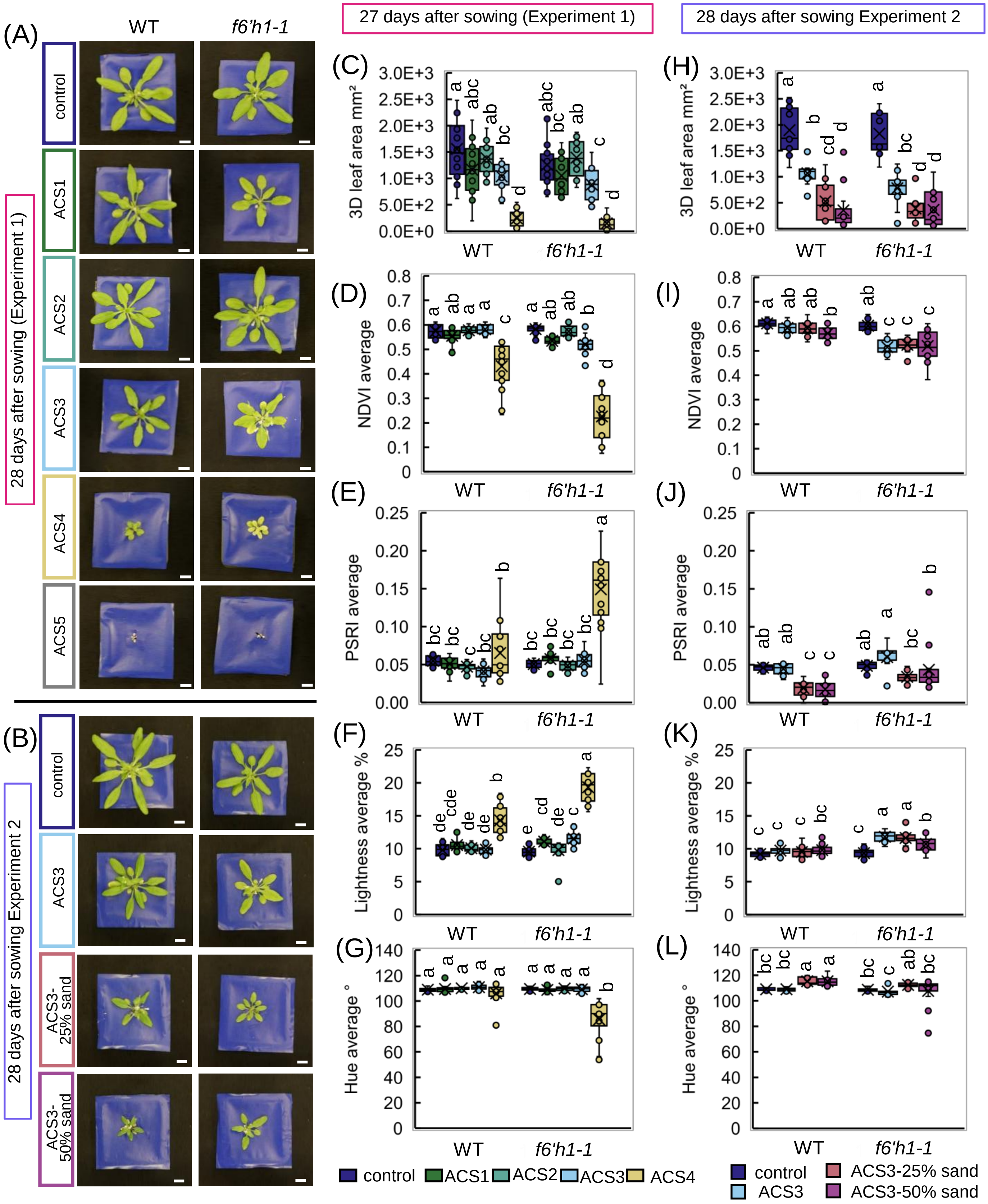

Supplemental Figure 8: Machine-Derived (PlantEye) Phenotypical Analysis of Wild Type (WT) and *f6'h1-1* in Seven Alkaline Calcareous Soil (ACS) Conditions 27/28 Days After Sowing (DAS).

A. thaliana wildtype (WT) and the coumarin deficient mutant *f6'h1-1 were* grown in seven alkaline calcareous soil (ACS) conditions with varying pH values in two experiments to determine an intermediate condition differentiating the lines. **A:** Plants in ACS1-5 (experiment 1) 28 days after sowing. Scale bar =1 cm. **B:** Plants in ACS3, ACS3-25% sand and ACS3 50%-sand (experiment 2) 28 days after sowing. Scale bar = 1 cm. **C-G:** 3D leaf area, normalized difference vegetation index (NDVI) average, plant senescence reflectance index (PSRI) average, lightness average and hue average in experiment 1 27 days after sowing determined with the PlantEye. Conditions from left to right were control and ACS1-ACS4 **H-L:** 3D leaf area, normalized difference vegetation index (NDVI) average, plant senescence reflectance index (PSRI) average, lightness average and hue average in experiment 2 28 days after sowing determined with the PlantEye. Conditions were from left to right control, ACS3, ACS3-25% sand and ACS3-50% sand. Letters indicate statistical significance. N=9-16 plants. Two-way ANOVA and Tukey-Test were performed in R. *p*=0.05. Graphs were created in Excel.

Supplemental Figure 9

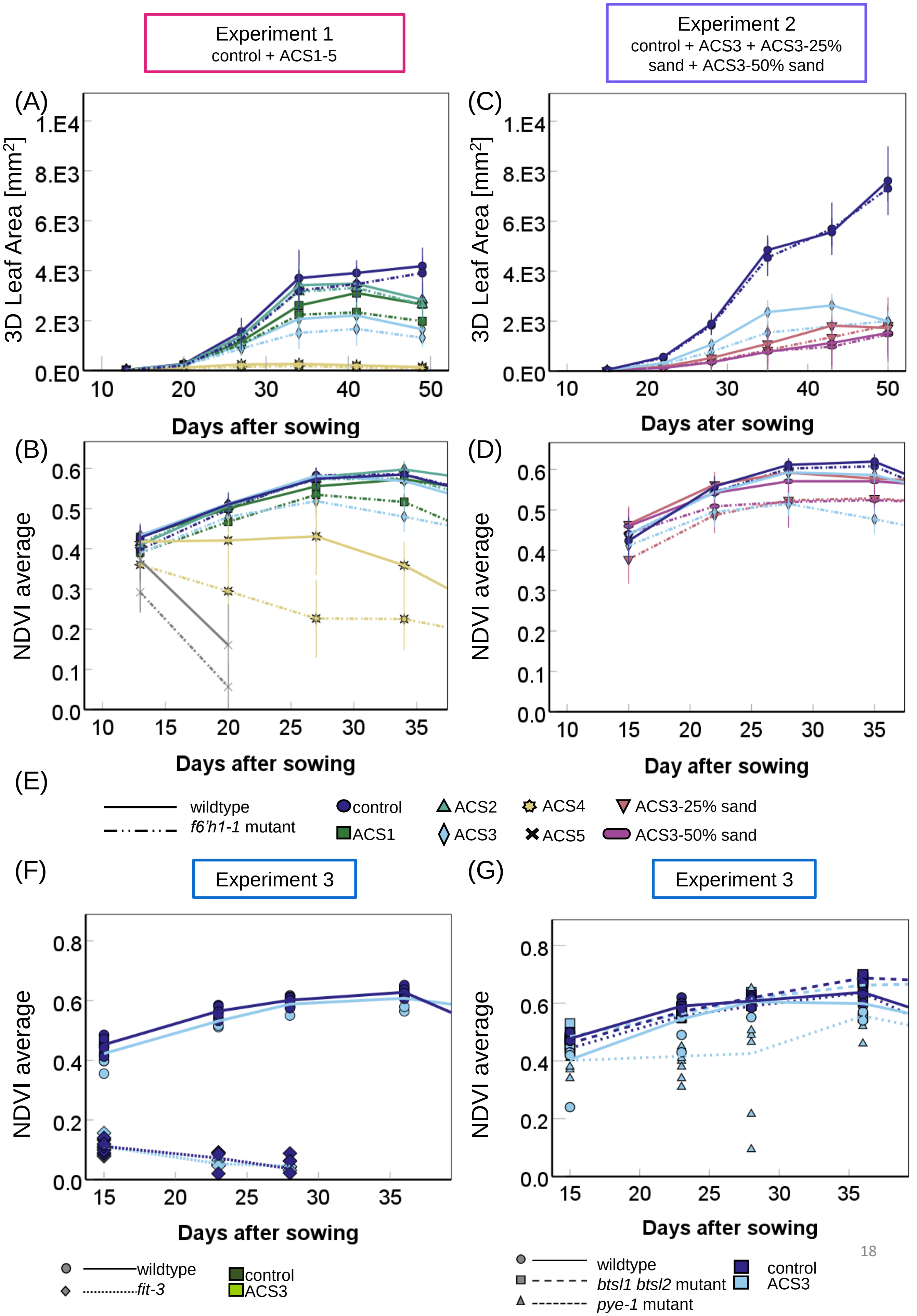

Supplemental Figure 9: Machine-Derived (PlantEye) Phenotypical Analysis of Wild Type (WT) and *f6'h1-1* in Seven Alkaline Calcareous Soil (ACS) Conditions During the Experiment

A. thaliana wildtype (WT) and the coumarin deficient mutant *f6'h1-1* were grown in seven alkaline calcareous soil (ACS) conditions with varying pH values in two experiments to determine an intermediate condition differentiating the lines. **A:** 3D leaf area and **B:** normalized difference vegetation index (NDVI) average of plants in ACS1-5 (experiment 1) between 15 and 50 (3D Leaf Area)/ 35 (NDVI average) days after sowing. **C:** 3D leaf area and **D:** NDVI average of plants in ACS3, ACS3-25% sand and ACS3 50%-sand (experiment 2) between 15 and 50 (3D leaf area)/ 35 (NDVI average) days after sowing. 3D leaf area and NDVI average were determined with the PlantEye. N=9-16 plants for 15 to 42 days after sowing and N= 6-8 plants for 50 days after sowing. Mean +/- standard deviation is shown. Graphs were created in SPSS. Measurement for NDVI later than 35 days after sowing were not included due shoots formation disturbing the results. **E:** Legend for A-D, solid line = WT, dashed line = *f6'h1-1* mutant. Different symbols and colors represent soil conditions used. **F:** Development of the NDVI of WT and *fit-3* mutant in control soil and ACS3 between 15 and 35 days after sowing. N= 3-8 plants**. G**: Development of the NDVI of WT, *btsl1 btsl2* mutant and *pye-1* mutant in control soil and ACS3 between 15 and 35 days after sowing. Single data points and interpolation line shown. Graphs were created in SPSS.N= 5-8 plans

Supplemental Table 1: All Phenotypic Data Collected Manually and Machine-Aided (PlantEye) for Correlation Analysis. - *Note that the full table must be provided as Excel Table that cannot be saved as a pdf; this page is a placeholder*

Tissue used for PlantEye and Phenospex measurement:					inflorescence stem	whole plant	whole plant	whole plant	whole plant	whole pl
Days after sowing:					depending on plant	13	13	13	13	
Experiment	Line	Treatment	Plant number	Block ID	Flowering time [days after sowing]	Manual rosette area [mm²]	Manual rosette convex hull [mm²]	Rosette diameter [cm]	3D Leaf Area mm²	Canopy L
1	wildtype	control	1	140	29			0.586	11.880	
1	wildtype	control	2	141	26			1.214	37.344	
1	wildtype	control	3	142	26			0.861	23.140	
1	wildtype	control	4	143	25	30.709	49.168	0.894	22.746	
1	wildtype	control	5	144	24			1.252	36.751	
1	wildtype	control	6	145	24			0.973	21.444	
1	wildtype	control	7	146	24			0.940	18.547	
1	wildtype	control	8	147	29			0.748	15.402	
1	wildtype	control	9	148	29			0.541	9.814	
1	wildtype	control	10	149	25	50.088	89.530	1.270	12.934	
1	wildtype	control	11	150	24			1.422	60.429	
1	wildtype	control	12	151	26			1.295	51.906	
1	wildtype	control	13	152	31					
1	wildtype	control	14	153	24	41.363	63.650	1.069	29.913	
1	wildtype	control	15	154	23			0.799	16.081	
1	wildtype	control	16	155	27			1.156	19.885	
1	wildtype	ACS1	1	156	35					
1	wildtype	ACS1	2	157	28			0.779	21.934	
1	wildtype	ACS1	3	158	29			0.654	14.012	
1	wildtype	ACS1	4	159	26			0.926	22.759	
1	wildtype	ACS1	5	160	27			1.291	37.385	
1	wildtype	ACS1	6	161	28	21.341	28.374	0.661	14.270	
1	wildtype	ACS1	7	162	31			0.683	12.001	4550000[illegible]
1	wildtype	ACS1	8	163	24	55.037	97.748	1.264	32.946	
1	wildtype	ACS1	9	164	29			1.008	26.327	
1	wildtype	ACS1	10	165	33					
1	wildtype	ACS1	11	166	29			0.970	21.714	
1	wildtype	ACS1	12	167	26			1.012	30.188	
1	wildtype	ACS1	13	168	24	63.414	93.744	1.306	41.290	
1	wildtype	ACS1	14	169	28			0.475	10.045	
1	wildtype	ACS1	15	170	24			1.393	45.914	
1	wildtype	ACS1	16	171	28			0.856	19.414	
1	wildtype	ACS3	1	172	30			0.956	16.728	
1	wildtype	ACS3	2	173	27			1.307	45.292	
1	wildtype	ACS3	3	174	24			1.072	12.904	
1	wildtype	ACS3	4	175	24			1.369	48.614	
1	wildtype	ACS3	5	176	28			0.592	26.599	
1	wildtype	ACS3	6	177	31			0.558	11.683	
1	wildtype	ACS3	7	178	28	44.405	66.831	1.006	25.942	
1	wildtype	ACS3	8	179	28	31.646	43.377	0.827	14.679	
1	wildtype	ACS3	9	180	25			1.127	32.985	
1	wildtype	ACS3	10	181	25			1.227	33.141	
1	wildtype	ACS3	11	182	27	37.137	60.119	0.975	20.000	
1	wildtype	ACS3	12	183	30			0.843	22.954	
1	wildtype	ACS3	13	184	24			0.671	15.948	
1	wildtype	ACS3	14	185	27			1.040	31.977	
1	wildtype	ACS3	15	186	27			1.008	26.409	
1	wildtype	ACS3	16	187	27			0.948	32.821	
1	wildtype	ACS2	1	188	28			0.560	9.174	
1	wildtype	ACS2	2	189	25			1.052	30.154	
1	wildtype	ACS2	3	190	26			1.047	25.608	
1	wildtype	ACS2	4	191	28			1.000	35.162	
1	wildtype	ACS2	5	192	28	31.717	54.952	0.951	20.100	
1	wildtype	ACS2	6	193	26	29.195	47.558	0.882	15.258	
1	wildtype	ACS2	7	194	27			0.861	15.651	
1	wildtype	ACS2	8	195	28					
1	wildtype	ACS2	9	196	25			0.883	19.904	
1	wildtype	ACS2	10	197	25	39.184	59.186	0.884	26.257	

Supplemental Table 2: Results of Correlation Analysis All Manually Determined and All Machine-Derived PlantEye Parameters.

Data of twelve suitable manual and 20 machine-derived PlantEye parameters for A. thaliana wildtype (WT) and its coumarin-deficient mutant *f6'h1-1* under control and up to seven alkaline calcareous soil (ACS) conditions, representing a scale of differing pH values from pH 6.2 (control) up to 8.3 (severe ACS) were recorded during two experiments and subjected to correlation analysis.Plant weight, rosette fresh weight and pigment contents were determined 38 days after sowing. Plant height was determined 44 days after sowing. Rosette diameter, manual rosette area and manual rosette convex hull were measured weekly during six weeks. N depended on parameters, if several time points were measured, plants were measured repeatedly. N (plant weight, rosette fresh weight, plant height) =24 plants, N (Rosette diameter) =799 data points, N (manual rosette area, manual convex hull area) =179 data point, N (Number of siliques and side branches) = 61 data points, N (pigment contents) = 23 datapoints. Only rosettes were used for chlorophyll content measurement in acetone. For details on parameters see materials and methods section. Spearman Rho Correlation was done in SPSS. Significant correlations are marked (* <0.05, ** <0.01). This table is equal to "Supplemental Table 2.docx"

Supplemental Table 2 (1/2)

	Rosette diameter [cm]	Manual rosette area [mm^2]	Manual rosette convex hull [mm^2]	Plant fresh weight [mg]	Rosette fresh weight [mg]	Plant height [cm]	Number of siliques	Number of side branches	Chlorophyll a [µg/mg FW]	Chlorophyll b [µg/mg FW]	Carotenoids [µg/mg FW]	Chlorophyll a+b [µg/mg FW]
3D Leaf Area mm^2	0.981**	0.992**	0.976**	0.766**	0.770**	0.478*	0.818**	0.144	-0.022	0.097	-0.242	-0.003
Canopy Light Penetration Depth mm	0.838**	0.915**	0.888**	0.698**	0.676**	0.744**	0.883**	0.435**	-0.054	0.043	-0.296	-0.036
Convex Hull Area Coverage %	-0.750**	-0.762**	-0.755**	-0.083	-0.093	-0.646**	-0.666**	-0.095	-0.529**	-0.544**	-0.300	-0.522*
Convex Hull Area mm^2	0.971**	0.969**	0.951**	0.731**	0.770**	0.608**	0.926**	0.169	0.021	0.141	-0.218	0.041
Convex Hull Aspect Ratio	0.285**	0.107	0.144	0.098	-0.174	0.042	-0.250	-0.100	0.249	0.264	0.278	0.263
Convex Hull Circumference mm	0.969**	0.966**	0.949**	0.675**	0.779**	0.616**	0.923**	0.153	0.033	0.157	-0.210	0.052
Convex Hull Maximum Width mm	0.964**	0.956**	0.937**	0.630**	0.777**	0.633**	0.913**	0.150	0.025	0.136	-0.211	0.040
Digital Biomass mm^3	0.897**	0.973**	0.951**	0.788**	0.751**	0.519**	0.906**	0.231	-0.056	0.060	-0.286	-0.036
GLI Average	0.712**	0.552**	0.591**	0.691**	0.750**	0.229	0.575**	0.228	0.001	0.072	-0.277	0.015
GLI [-0.2:0] %	-0.430**	-0.274**	-0.319**	-0.339	-0.605**	-0.535**	-0.772**	-0.126	-0.059	-0.098	0.244	-0.075
GLI [-1:-0.2] %	0.074*	0.042	0.018			-0.256	-0.175	-0.075				
GLI [0.15:0.3] %	-0.463**	-0.389**	-0.449**	-0.531**	-0.639**	-0.540**	0.468**	0.494**	0.257	0.164	0.520*	0.238
GLI [0.3:0.6] %	0.691**	0.556**	0.602**	0.688**	0.728**	0.306	0.536**	0.215	-0.091	-0.010	-0.332	-0.079
GLI [0.6:1] %	0.654**	0.613**	0.574**	0.665**	0.671**	0.365	0.318*	0.381**	0.238	0.302	-0.027	0.249
GLI [0:0.15] %	-0.705**	-0.556**	-0.587**	-0.661**	-0.763**	0.073	-0.310*	-0.112	-0.031	-0.105	0.205	-0.041
Hue Average °	-0.259**	-0.325**	-0.297**	-0.353	-0.210	0.495*	0.841**	0.141	0.695**	0.578**	0.603**	0.687**
Hue [0:25] %	-0.433**	-0.296**	-0.338**	-0.274	-0.581**	-0.511*	-0.786**	-0.138	-0.067	-0.106	0.233	-0.085
Hue [100:125] %	0.635**	0.481**	0.520**	-0.041	0.263	-0.113	0.525**	0.368**	0.557**	0.478*	0.343	0.547**
Hue [125:360] %	-0.517**	-0.411**	-0.407**	-0.610**	-0.643**	0.508*	0.897**	0.196	0.351	0.244	0.503*	0.340
Hue [25:50] %	-0.299**	-0.089	-0.146	-0.476*	-0.550**	-0.637**	-0.728**	-0.147	-0.119	-0.141	0.124	-0.130
Hue [50:75] %	-0.297**	-0.133	-0.196**	-0.463*	-0.561**	-0.588**	-0.377**	0.259*	-0.449*	-0.444*	-0.108	-0.456*
Hue [75:100] %	-0.268**	-0.240**	-0.244**	0.500*	0.445*	0.270	0.510**	0.268*	-0.657**	-0.533**	-0.764**	-0.633**
Lightness Average %	-0.641**	-0.698**	-0.706**	-0.458*	-0.589**	0.045	-0.206	-0.526**	-0.544**	-0.529**	-0.343	-0.533**
Lightness [0:0] %												
Lightness [0:25] %	0.448**	0.399**	0.430**	0.283	0.503*	-0.487*	0.186	0.347**	0.409	0.440*	0.171	0.420*
Lightness [100:100] %												
Lightness [25:50] %	-0.441**	-0.395**	-0.426**	-0.286	-0.503*	0.487*	-0.186	-0.347**	-0.409	-0.440*	-0.171	-0.420*
Lightness [50:75] %	-0.004	-0.051	-0.049	0.351	0.259	0.226	0.249	-0.272*	-0.269	-0.236	-0.103	-0.281
Lightness [75:100] %	0.017	0.065	0.100				0.246	0.113				
NDVI Average	0.675**	0.548**	0.579**	0.244	0.552**	-0.344	0.248	0.326*	0.586**	0.570**	0.321	0.579**
NDVI [-1:0] %	-0.309**	-0.280**	-0.307**	-0.048	-0.101	-0.051	-0.421**	-0.178	-0.458*	-0.411	-0.325	-0.460*

Supplemental Table 2 (2/2)

	Rosette diameter [cm]	Manual rosette area [mm^2]	Manual rosette convex hull [mm^2]	Plant fresh weight [mg]	Rosette fresh weight [mg]	Plant height [cm]	Number of siliques	Number of side branches	Chlorophyll a [µg/mg FW]	Chlorophyll b [µg/mg FW]	Carotenoids [µg/mg FW]	Chlorophyll a+b [µg/mg FW]
NDVI [0.15:0.3] %	-0.390**	-0.214**	-0.264**	-0.155	-0.543**	0.756**	0.538**	0.144	-0.504*	-0.490*	-0.245	-0.502*
NDVI [0.3:0.45] %	-0.663**	-0.559**	-0.586**	-0.263	-0.411*	0.396	0.525**	0.215	-0.614**	-0.585**	-0.425*	-0.601**
NDVI [0.45:0.6] %	0.002	0.033	0.067	-0.360	-0.513*	-0.156	0.351**	0.194	-0.450*	-0.417*	-0.247	-0.430*
NDVI [0.6:1] %	0.823**	0.792**	0.800**	0.326	0.565**	-0.185	0.133	0.492**	0.598**	0.573**	0.356	0.587**
NDVI [0:0.15] %	-0.326**	-0.170*	-0.230**	-0.325	-0.570**	0.119	-0.452**	-0.138	-0.381	-0.401	-0.107	-0.384
NPCI Average	0.539**	0.494**	0.483**	0.662**	0.677**	-0.136	-0.664**	0.027	-0.444*	-0.318	-0.708**	-0.419*
NPCI [-0.2:0] %	-0.519**	-0.407**	-0.410**	-0.678**	-0.688**	0.515*	0.891**	0.194	0.353	0.251	0.533**	0.336
NPCI [-1:-0.2] %	0.326**	0.339**	0.350**	-0.070	-0.013	-0.197	0.797**	0.180	0.337	0.216	0.389	0.324
NPCI [0.2:0.4] %	0.423**	0.471**	0.464**	0.631**	0.631**	0.135	-0.234	-0.122	-0.520*	-0.398	-0.744**	-0.496*
NPCI [0.4:0.6] %	0.681**	0.744**	0.699**	0.622**	0.719**	-0.030	-0.052	0.313*	0.014	0.140	-0.164	0.035
NPCI [0.6:1] %	0.676**	0.704**	0.654**	0.686**	0.474*	0.556**	0.271*	0.539**	0.192	0.221	-0.249	0.211
NPCI [0:0.2] %	0.138**	-0.007	0.012	-0.567**	-0.530**	-0.468*	-0.618**	0.067	0.412	0.296	0.647**	0.386
PSRI Average	0.251**	0.298**	0.271**	0.457*	0.422*	-0.359	-0.796**	-0.178	-0.673**	-0.532**	-0.715**	-0.652**
PSRI [-0.2:0] %	-0.494**	-0.388**	-0.391**	-0.648**	-0.621**	0.462*	0.874**	0.192	0.418*	0.300	0.588**	0.396
PSRI [-0.4:-0.2] %	-0.440**	-0.347**	-0.338**	-0.043	-0.129	0.720**	0.895**	0.185	-0.141	-0.210	-0.103	-0.132
PSRI [-0.8:-0.4] %	0.366**	0.382**	0.388**	0.316	0.139	0.294	0.885**	0.155	-0.146	-0.007	-0.150	-0.113
PSRI [-4:-0.8] %	0.137**	0.146	0.140				0.436**	-0.016				
PSRI [0.2:4] %	0.025	0.160*	0.103	-0.356	-0.460*	-0.364	-0.637**	-0.188	-0.452*	-0.436*	-0.144	-0.453*
PSRI [0:0.2] %	0.632**	0.473**	0.514**	0.583**	0.618**	-0.092	0.021	0.313*	-0.049	0.048	-0.329	-0.029
Plant Height Averaged mm	0.846**	0.913**	0.888**	0.693**	0.708**	0.744**	0.883**	0.439**	-0.102	0.018	-0.367	-0.080
Plant Height Max mm	0.910**	0.917**	0.892**	0.674**	0.703**	0.743**	0.889**	0.407**	-0.081	0.014	-0.337	-0.060
Projected Leaf Area mm²	0.980**	0.991**	0.976**	0.768**	0.770**	0.477*	0.819**	0.144	-0.015	0.107	-0.234	0.004
Saturation Average %	0.758**	0.643**	0.672**	0.748**	0.789**	0.075	0.392**	0.178	-0.212	-0.103	-0.492*	-0.190
Saturation [0:0] %												
Saturation [0:25] %	-0.733**	-0.606**	-0.634**	-0.783**	-0.810**	0.306	-0.316*	-0.205	0.193	0.091	0.428*	0.177
Saturation [100:100] %	0.215**	0.245**	0.238**			0.413*	0.361**	0.382**				
Saturation [25:50] %	0.498**	0.385**	0.414**	-0.139	-0.090	-0.752**	0.197	0.276*	-0.435*	-0.439*	-0.089	-0.450*
Saturation [50:75] %	0.800**	0.770**	0.786**	0.692**	0.731**	0.574**	0.475**	0.198	-0.241	-0.140	-0.535**	-0.218
Saturation [75:100] %	0.702**	0.742**	0.695**	0.596**	0.738**	0.466*	0.471**	0.522**	0.193	0.239	-0.158	0.205
Surface Angle Average °	-0.587**	-0.672**	-0.636**	-0.129	-0.491*	-0.265	0.011	-0.394**	0.053	0.019	0.372	0.044
Voxel Volume Total mm³	0.980**	0.990**	0.974**	0.764**	0.770**	0.486*	0.877**	0.164	-0.016	0.107	-0.238	0.003

** Correlation is significant at the 0.01 level (2-tailed).

* Correlation is significant at the 0.05 level (2-tailed).

Supplemental Table 3**: All Machine-Derived Data for Phenotypic Analysis Using Known Regulatory Iron Homeostasis Mutants**. - *Note that the full table must be provided as Excel Table that cannot be saved as a pdf; this page is a placeholder*

Block ID	Days after s(	Plant tissue scanned	Experiment	Line	Treatment	3D Leaf Area	Canopy Light Penet(	Convex Hull	Convex Hull
633:1:1	15	whole plant	3	WT	control	122.519	4.120	49.319	203.037
634:1:1	15	whole plant	3	WT	control	84.925	1.965	44.723	167.671
635:1:1	15	whole plant	3	WT	control	28.141	2.053	60.400	40.230
636:1:1	15	whole plant	3	WT	control	59.854	1.978	40.248	119.699
637:1:1	15	whole plant	3	WT	control	121.204	2.981	44.549	227.072
638:1:1	15	whole plant	3	WT	control	104.027	2.902	46.587	183.780
639:1:1	15	whole plant	3	WT	control	55.213	1.955	42.274	114.751
640:1:1	15	whole plant	3	WT	control	65.312	3.456	45.194	120.514
641:1:1	15	whole plant	3	WT	ACS3	20.568	1.629	55.803	31.589
642:1:1	15	whole plant	3	WT	ACS3	48.125	1.985	59.787	69.659
643:1:1	15	whole plant	3	WT	ACS3	91.043	3.081	36.206	212.491
643:1:1	15	whole plant	3	WT	ACS3	53.534	1.803	58.711	74.520
644:1:1	15	whole plant	3	WT	ACS3	43.434	1.311	63.820	56.700
645:1:1	15	whole plant	3	WT	ACS3	63.109	2.105	54.272	100.981
646:1:1	15	whole plant	3	WT	ACS3	48.882	2.192	61.576	71.281
648:1:1	15	whole plant	3	WT	ACS3	24.075	1.375	55.648	38.969
857:1:1	15	whole plant	3	fit-3	control	44.912	2.808	62.911	58.230
859:1:1	15	whole plant	3	fit-3	control	8.997	0.000	45.951	18.180
860:1:1	15	whole plant	3	fit-3	control	23.040	1.863	54.620	32.310
861:1:1	15	whole plant	3	fit-3	control	20.085	1.472	47.632	35.550
862:1:1	15	whole plant	3	fit-3	control	20.083	1.533	74.985	22.320
863:1:1	15	whole plant	3	fit-3	control	36.703	3.225	44.881	92.340
864:1:1	15	whole plant	3	fit-3	control	26.902	1.227	50.097	47.070
865:1:1	15	whole plant	3	fit-3	ACS3	10.758	0.000	60.083	15.120
866:1:1	15	whole plant	3	fit-3	ACS3	21.698	1.521	49.252	38.340
867:1:1	15	whole plant	3	fit-3	ACS3	9.656	0.000	58.383	14.673
869:1:1	15	whole plant	3	fit-3	ACS3	12.818	0.178	58.668	19.800
870:1:1	15	whole plant	3	fit-3	ACS3	21.440	2.730	51.561	31.950
871:1:1	15	whole plant	3	fit-3	ACS3	18.216	2.042	65.546	23.041
872:1:1	15	whole plant	3	fit-3	ACS3	25.650	2.475	54.400	38.879
633:1:1	23	whole plant	3	WT	control	1253.250	5.350	39.772	2547.180
634:1:1	23	whole plant	3	WT	control	967.661	5.394	37.228	2109.510
635:1:1	23	whole plant	3	WT	control	517.726	5.752	39.320	1065.060
636:1:1	23	whole plant	3	WT	control	851.147	6.865	43.189	1657.890
637:1:1	23	whole plant	3	WT	control	1475.460	4.860	37.389	3172.685
638:1:1	23	whole plant	3	WT	control	1201.295	7.618	39.962	2406.060
639:1:1	23	whole plant	3	WT	control	851.617	6.051	39.732	1753.830
640:1:1	23	whole plant	3	WT	control	877.918	9.594	34.876	2073.595
641:1:1	23	whole plant	3	WT	ACS3	56.429	2.287	72.306	62.730
642:1:1	23	whole plant	3	WT	ACS3	353.426	5.253	49.451	593.280
643:1:1	23	whole plant	3	WT	ACS3	246.406	5.077	71.036	262.891
644:1:1	23	whole plant	3	WT	ACS3	260.579	4.400	52.134	384.301
645:1:1	23	whole plant	3	WT	ACS3	437.974	3.272	54.242	644.943
646:1:1	23	whole plant	3	WT	ACS3	273.122	2.736	63.410	355.142
648:1:1	23	whole plant	3	WT	ACS3	187.920	1.609	54.313	304.922
857:1:1	23	whole plant	3	fit-3	control	17.788	1.527	64.946	22.591
860:1:1	23	whole plant	3	fit-3	control	19.387	1.350	72.870	23.310
862:1:1	23	whole plant	3	fit-3	control	12.175	0.000	62.130	16.560
864:1:1	23	whole plant	3	fit-3	control	28.063	0.628	54.087	48.688
866:1:1	23	whole plant	3	fit-3	ACS3	24.362	1.451	55.879	36.811
870:1:1	23	whole plant	3	fit-3	ACS3	18.767	1.913	51.014	31.590
871:1:1	23	whole plant	3	fit-3	ACS3	13.472	1.800	51.073	21.330
872:1:1	23	whole plant	3	fit-3	ACS3	26.012	2.208	61.645	36.181
633:1:1	28	whole plant	3	WT	control	3550.630	20.444	34.104	8610.750
634:1:1	28	whole plant	3	WT	control	2911.750	16.276	33.914	7070.400
635:1:1	28	whole plant	3	WT	control	1672.005	16.684	32.852	4042.625
636:1:1	28	whole plant	3	WT	control	2447.380	20.530	31.915	6093.540
637:1:1	28	whole plant	3	WT	control	3570.415	41.712	29.887	9503.375
638:1:1	28	whole plant	3	WT	control	2907.185	28.521	31.550	7594.385
639:1:1	28	whole plant	3	WT	control	2280.685	58.055	38.469	4445.010
640:1:1	28	whole plant	3	WT	control	2203.840	53.505	33.692	5241.055
641:1:1	28	whole plant	3	WT	ACS3	188.974	5.570	60.861	226.711
642:1:1	28	whole plant	3	WT	ACS3	1110.210	5.978	44.698	2050.920
643:1:1	28	whole plant	3	WT	ACS3	885.067	4.590	58.051	1303.200
644:1:1	28	whole plant	3	WT	ACS3	596.138	22.129	39.842	1228.770
[illegible]	[illegible]	[illegible]	[illegible]	[illegible]	[illegible]	[illegible]	[illegible]	[illegible]	[illegible]

Supplemental Methods

For this study, three independent experiments were conducted (Supplemental Figure 1) In the first two experiments different artificial alkaline calcareous soil conditions were tested using the wildtype (Col-0) and the coumarin deficient mutant *f6'h1-1*. The data were used to analyse correlation of machine (PlantEye) data and manual data. In the third experiment, the growth of the iron homeostasis mutants *fit-3, pye-1, btsl1 btsl2* was tested in one alkaline calcareous condition.

Plant Material

Lines of Arabidopsis thaliana (L.) Heynh. were multiplied in parallel at Heinrich Heine University. They were wildtype (WT, Col-0) and four mutants in Col-0 background, *f6'h1-1* (Schmid et al. 2014) , *fit-3* (Jakoby et al. 2004), *pye-1* (Long et al. 2010), and *btsl1 btsl2* (Rodríguez-Celma et al. 2019).

Plant Growth

Seeds were surface sterilized by incubation in 6% NaOCl and 0.1% TritonX100 for seven minutes followed by five times washing with ultrapure water. Seeds were stratified in darkness at 4°C on ½ Hoagland plates (macronutrients: 0.75 mM $MgSO_4$, 0.5 mM KH_2PO_4, 1.25 mM KNO_3, 1.5 mM $Ca(NO_3)_2$; micronutrients: 50 µM KCl; 50 µM H_3BO_3; 10 µM $MnSO_4$; 2 µM $ZnSO_4$; 1.5 µM $CuSO_4$; 0.075 µM; $(NH_4)_6Mo_7O_{24}$; 50 µM FeNaEDTA; 1% sucrose (w/v); 1.4% plant agar (w/v) pH 5.8-6.0), before being grown in near to vertical position for eight days (CU-36L4/D, CLF Plant Climatics, 16 h light (in average 135 $\mu mol \cdot m^{-2} \cdot s^{-1}$, fluorescent tube light, ecolux F17 T8 17W 4100K), 21°C in light, 19°C in darkness and 50% relative humidity). Day of placing the plates in light was considered as day of sowing.

On the eighth day plants were transferred to soil. Different soil conditions were prepared (Supplemental Figure 2). The basis for all conditions was peat-based growth substrate (Floraton 1, Floragard, Oldenburg, Germany). It was baked at 80°C over night in a drying oven (Memmert, Schwabach, Deutschland). In all conditions 400 ml deionised water (dH_2O) and 20 g vermiculite (Agrivermiculite Floragard, Oldenburg, Germany) were added per litre dry soil. Specific amounts of $CaCO_3$ (AppliChem, Darmstadt, Germany), $NaHCO_3$ (Fisher Scientific, Hampton, USA) and three-times washed sand (PROBAU Quarzsand eco, Bauhaus, Mannheim, Germany) were added. The soil was then mixed by hand and incubated for 30 minutes before the pH was determined. The soil pH was determined as follows: to 15 g wet soil (5 g soil dry weight) deionised water was added to reach 50 ml in a falcon tube. This was rotated for 30 min with 20 rotations per min and centrifuged for 10 min at 4000 *g* and 20°C (Heraeus Multifuge X1R, Thermo Fischer Scientific, Hampton, USA). The emerging supernatant was filtered through paper filter (Folded filters, 322345, Schleicher&Schuell, Dassel, Germany) and the pH was determined with a pH electrode. Two samples per condition were taken and the average was calculated.

In short the soil conditions were control (0 g/l $CaCO_{3,}$ 0 g/l $NaHCO_3$, 0 % sand v/v), ACS1 (6 g/l $CaCO_{3,}$ 3 g/l $NaHCO_3$, 0 % sand v/v), ACS2 (8 g/l $CaCO_{3,}$ 4 g/l $NaHCO_3$, 0 % sand v/v), ACS3 (8 g/l $CaCO_{3,}$ 4 g/l $NaHCO_3$, 0 % sand v/v), ACS4 (32 g/l $CaCO_{3,}$ 14 g/l $NaHCO_3$, 0 % sand v/v), ACS5 (30 g/l $CaCO_{3,}$ 21 g/l $NaHCO_3$, 0 % sand v/v), ACS3-25% sand (8 g/l $CaCO_{3,}$ 4 g/l $NaHCO_3$, 25 % sand v/v) and ACS3-50% sand (8 g/l $CaCO_{3,}$ 4 g/l $NaHCO_3$, 50 % sand v/v). In conditions ACS2, ACS4 and ACS5 there was a top soil consisting of 20 g control soil at the top of the pot. Conditions ACS1 and ACS3 were watered with a $NaHCO_3$- solution between the third and sixth week. In total each plant in ACS1 received 1.304 g and in ACS3 1.567 g $NaHCO_3$ in that time with their water split between four irrigations.

Depending on the condition, a certain weight of soil was filled into pots (height 5.6 cm, length and width ca 5.5 cm): i) 120-130 g soil without sand, ii) 180-190 g soil with 25% sand and iii) 220-230 g soil with 50% sand. After the pots had been filled, they were covered with precut matt blue vinyl foil (article number: 10335 370, https://www.kreativplotter.de/plotterfolien/vinylfolie, Europe Warehouse GmbH &Co. KG, Wuppertal, Germany). The foil was cut into squares of 7.0-7.5 cm. The white foil that was attached to the blue foil was cut into ca 5.5 cm squares and stuck reversely into the middle of the sliced blue foil to reduce contact between the glue of the foil and the soil. Holes with 1 cm diameter were cut into the squares using a hollow punch before they were stuck onto the pots. Then seedlings were planted into the holes and in the first week the holes were closed with small patches of soil only leaving the plant out (Supplemental Figure 2).

Plants were grown in plant cabinets (PK520, Polyklima, Freising, Germany) with 16 h light (98-112 µmol·m^{-2}·s^{-1}, Polyklima True Daylight + LED), 21°C during light, and 19°C during darkness. Humidity was not controlled. In experiment 3, plants were moved to a walk-in growth chamber with 16 h light (80-120 µmol m^{-2}· s^{-1}, BX120c4, VAYOLA, Helsinki, Finland), 21°C day temperature, 19°C night temperature and 57% humidity after three weeks in soil instead of being kept in a growth cabinet. Eight pots fitted in one tray. Sixteen plants were grown per line and condition for experiments 1 and 2, eight for experiment 3. Trays were regularly rotated.

Phenotyping

Chlorophyll content

The chlorophyll content was determined from whole rosettes of plants from experiment 2. The rosettes were frozen and grinded in liquid nitrogen with a ceramics mortar. Up to 120 mg of the powder was used. Two millilitre ice cool 100 % acetone was added and it was mixed for two minutes. Afterwards, tubes were centrifuged at 15,000 *g* for 10 min. The absorption was measured at 470 nm, 642 nm and 662 nm (Shimadzu UV visible Spectrophotometer UVmini-1240, Duisburg, Germany and Hellma OS 104-OS cuvette, Müllheim, Germany). Depending on the amount of used plant material, the samples were diluted. The pigment contents in the supernatant were calculated using the following formulas (Hartmut K. Lichtenthaler, 1987):

Chlorophyll a: (µg/ml) = (11.24*A662 - 2.04*A642) * dilution

Chlorophyll b: (µg/ml) = (20.13*A642 - 4.19*A662) * dilution

Carotenoids: (µg/ml) = [(1000*A470-1.90*Chla - 63.14*Chlb)/214] * dilution.

These values were multiplied by 2 ml and divided by the used fresh weight.

Manual Parameters

Different parameters were manually determined (Supplemental Figure 3). These included the rosette diameter [cm], manual rosette area [mm^2], manual rosette convex hull [mm^2], plant fresh weight [mg], rosette fresh weight [mg], plant height [cm], number of siliques, number of side branches, flowering time [days after sowing].

In the case of small plants (14 and 21 days after sowing), the rosette diameters were determined with Image J (v. 1.53 t). Two or three straight lines, depending on the number of leaves, were drawn between the tips of opposite leaves and the average was calculated. The longest possible diameters without using a leaf for more than one diameter were measured. At the other time points, when the leaves were overlapping, the rosette diameter was determined with a ruler. Again, the three longest distances between opposite leaf tips were measured and the average calculated. For this, leaves were moved to be in straight lines if necessary.

The manual rosette area and manual convex hull area were determined for the first experiment with ImageJ. For the manual rosette area, a line was drawn closely around the edges of the rosette and the area within the line was determined. The manual convex hull was drawn as a polygon around the rosette with no angle bigger than 180°. Both were determined weekly.

The plant height was determined once after 44 days. It was measured from the blue foil to the tip of the longest inflorescences after they had been stretched in a straight line. On the same day, the plants were scanned with the PlantEye.

The flowering time was noted when the first inflorescence was longer than 1.5 cm.

In the first experiment, the number of siliques and side branches were counted with ImageJ from photos. Plants were placed between two glass panes for that. Only half of the available plants were used for counting siliques and side branches, while the other one was bagged for seed harvesting. In the second and third experiment, the siliques and branches were counted directly at the plants. In any case, siliques were counted if they were either elongated longer than the petals or had no petals at all. Side branches were counted if they had formed their own first visible leaf.

The plant fresh weight and rosette fresh weight were determined destructively using a scale (Secura225D, Sartorius Lob Instruments GmbH & Co. KG, Germany). Whole plants were scanned with the PlantEye, then shoots were removed and the remaining rosettes scanned again. Afterwards, the shoots and rosettes were immediately weighed to prevent water loss before weighing. For whole plant fresh weight, weight of shoots and rosette were added for each plant. Plants used here were the same plants as used for pigment content analysis and were afterwards no longer included in any measurements.
For the correlations, the manual rosette diameter and the PlantEye measurement were done maximum one day apart of each other, while all the other measurements were done immediately after each other.

Machine (PlantEye) Parameters

Plants were measured separately by placing the pots in a blue holder that would keep the plants at a fixed height and within the measured unit. Plants were either measured four times (Experiments 1 and 2) or twice (Experiment 3) per measurement as technical replicates and turned by 90° in between. To remove the background, the colour range from the hue values 200-360° (blue to purple) was removed from all images on which the calculation of parameters relies. The PlantEye measured the reflection of a laser (935-945 nm), red (624-634 nm), green (530-540 nm), blue (465-485 nm) and near-infrared (nir, 935-945 nm). The parameters are split into morphological parameters (Supplemental Figure 4) and spectral parameters (Supplemental Figure 5). The morphological parameters are the 3D leaf area [mm^2] (Area of the plants seen from above if it was flattened to ground), canopy light penetrations depth [mm] (how far laser reaches into the canopy), convex hull area [mm^2] (Area of a convex hull drawn around the plant), convex hull area coverage [%] (proportion of the convex hull covered by the plants), convex hull maximum width [mm] (longest straight length in the convex hull), convex hull aspect ratio [%] (Quotient of the convex hull maximum width and the perpendicular line to it at its midpoint), convex hull circumference [mm], plant height max [mm] (Distance from the pot height to the highest points of the plant), plant height averaged [mm] (highest point of is replaced by average height of the highest 10%), projected leaf area [mm^2] (area covered by plant seen from above), digital biomass [mm^3] (product of 3D leaf area and plant height averaged), surface angle average [°] (average angle of all triangles between points forming a plant) and voxel volume total [mm^3] (sum of all voxels representing the plant).
The spectral parameters are hue average [°] (colour value of the HSL, Hue-Saturation-Lightness colour space), saturation average [%] (value of HSL, grey 100% to pure colour 0%), lightness average [%] (value of HSL, white 100% to black 0%), greenness leaf index (GLI, (2·GREEN-RED-BLUE)/(RED+GREEN+BLUE)), normalized difference vegetation index (NDVI, (NIR-RED)/(NIR+RED)), normalized pigment chlorophyll index (NPCI, (RED-BLUE)/(RED+BLUE)) and plant senescence reflectance index (PSRI, (RED-BLUE)/NIR). For all these parameters the average per plant and the percentage of voxels within different (definable) ranges are calculated.
